# A Practical Framework for Incorporating Complex Survey Design in Bayesian Kernel Machine Regression

**DOI:** 10.3390/stats9030046

**Published:** 2026-04-23

**Authors:** Doreen Jehu-Appiah, Emmanuel Obeng-Gyasi

**Affiliations:** 1 Department of Built Environment, North Carolina A&T State University, Greensboro, NC 27411, USA; 2 Department of Computational Data Science and Engineering, North Carolina A&T State University, Greensboro, NC 27411, USA; 3 Environmental Health and Disease Laboratory, North Carolina A&T State University, Greensboro, NC 27411, USA

**Keywords:** Bayesian kernel machine regression, complex survey design, environmental exposure mixtures, informative sampling, population-representative inference, resampling methods

## Abstract

Large-scale population datasets are rarely generated via simple random sampling; instead, they reflect complex designs involving stratification, clustering, and unequal inclusion probabilities. While survey weights are provided to recover population-representative estimates, standard Bayesian Kernel Machine Regression (BKMR), a flexible nonlinear model for high-dimensional exposure mixtures, does not explicitly accommodate these design features. We present a simulation-based framework that evaluates performance under complex sampling by comparing two analytic strategies applied to identical survey-like data: (i) a naïve, unweighted BKMR implementation and (ii) a design-aware workflow that can be executed using existing software without modifying the BKMR algorithm itself. Finite populations are generated with correlated exposures and a known nonlinear data-generating function. Stratified two-stage cluster samples are then drawn under both non-informative and exposure-dependent (informative) selection mechanisms, with controlled intra-class correlation (ICC). The design-aware approach incorporates sampling weights through resampling of the dataset while preserving primary sampling unit structure, followed by standard BKMR fitting. Methods are evaluated using bias, interval width, and empirical 95% coverage relative to the known truth. Across simulation scenarios, naïve BKMR exhibits bias and systematic under-coverage under informative sampling, with empirical 95% coverage often dropping to approximately 0–40%, whereas the design-aware workflow improves coverage to approximately 40–60%, moving results closer to nominal levels. These findings provide a practical, implementation-ready strategy for integrating survey design considerations into BKMR analyses and delineate conditions under which accounting for sampling design affects inference. While the proposed approach improves inferential performance relative to naïve BKMR, it does not fully achieve nominal coverage, indicating that further methodological development is required for fully valid uncertainty quantification under complex survey designs.

## 1. Introduction

National health surveys are typically conducted under complex probability sampling designs that incorporate stratification, multi-stage clustering through primary sampling units, and unequal inclusion probabilities. Sampling weights are provided to enable valid population-level inference under these designs. However, despite the widespread use of BKMR in environmental mixture studies, there is currently no standard, implementation-ready approach for incorporating complex survey design into BKMR, creating a critical gap between methodological practice and population-representative inference. Failure to account for complex survey design, including unequal selection probabilities and within-cluster dependence, produces biased estimates and anticonservative uncertainty, because key sources of design-induced variability are not properly propagated into the analysis [1–4].

This matters in environmental epidemiology, where BKMR is widely used to learn non-linear and interactive exposure–response relationships in mixtures [5]. Recent mixture analyses of NHANES biomarker data, such as Jehu-Appiah and Obeng-Gyasi [6], have demonstrated that combined exposures to metals, PFAS, phthalates, and plasticizers jointly influence cardiovascular risk. However, these studies typically apply BKMR under an unweighted framework, potentially overlooking survey weights and clustering that affect population-level inference. Current BKMR implementations, however, do not natively accept survey weights or replicate-weight variance estimators, so analysts often fit unweighted BKMR and label results exploratory, a practice that can misstate both effect sizes and their uncertainty.

Our aim is to show how conclusions change when the same survey-like data are analyzed (i) with naïve, unweighted BKMR versus (ii) a design-aware workflow that is feasible with today’s tools. We build a simulation lab that mimics real survey conditions: correlated exposures, stratified two-stage cluster sampling, informative within-cluster selection, and intra-class correlation (ICC). For each scenario we fit standard BKMR and compare it to a weight-aware resampling approach that redraws the analysis set in proportion to survey weights (and respects PSUs) before running. We evaluate performance against the known truth from the data-generating process using simple metrics like bias, interval width, and coverage.

Methodologically, our approach sits between two established views. The design-based view emphasizes using weights (and, when available, replicate weights) so inference targets the population and uncertainty reflects the sampling design [7]. The model-based view (e.g., multilevel regression and post-stratification, MRP) argues that if a model captures the factors that drive selection, then post-stratifying predictions can also recover population quantities [1]. Both perspectives warn that ignoring the design risks biased, over-confident results—a concern that we explicitly demonstrate and quantify in this work.

Rather than modifying BKMR’s likelihood or introducing a new estimator, we demonstrate that weight-proportional resampling with PSU preservation enables BKMR to approximate a design-weighted empirical distribution while accounting for clustering-induced uncertainty. The methodological contribution is therefore not a new survey estimator in general, but a BKMR-compatible, implementation-ready workflow that brings survey-aware resampling, replication-based uncertainty, and mixture-specific summaries into a single framework.

### Background and Related Work

Population health surveys typically use complex sampling designs involving stratification, clustering, and unequal selection probabilities, with weights used to obtain population-representative estimates. Ignoring these design features can lead to biased point estimates and underestimated uncertainty by failing to account for within-cluster dependence and unequal inclusion [8–10]. These concerns are particularly acute in environmental epidemiology, where exposure mixtures frequently exhibit non-linearity and interactions. For example, the flexible modelling technique BKMR, is now widely adopted for mixtures analysis [5,11,12]. Yet, mainstream implementations of BKMR often lack native support for complex design features (such as survey weights, PSUs, or replicate-weights), motivating many analysts to run unweighted fits exploratorily with attendant risks of bias and under-coverage [13–15].

Several methodological strands suggest principled remedies for incorporating survey design features into Bayesian mixture analyses. A model-based route, often called multilevel regression and post-stratification (MRP), seeks to encode design drivers (for example, geography, strata, demographic covariates) within the model and then post-stratify predictions to the population [1]. When the model sufficiently captures the structure of selection and population heterogeneity, MRP can deliver design-consistent population estimates. However, in realistic survey settings the design and the exposure mechanism may both be complex and only partially measured, which limits full applicability of standard MRP models to fully correct for unequal inclusion [16,17]. Recent innovations, such as Multilevel Regression and Post-stratification with Weights (MRPW), explicitly integrate sampling weights into Bayesian hierarchical frameworks to enhance representativeness and reduce model misspecification [18]. Despite these advances, most implementations remain computationally demanding and require substantial auxiliary information that may not always be available in practice.

A second route employs replicate-weight and bootstrapping methods to achieve design-based variance estimation under clustering and unequal probabilities. These methods, such as Balanced Repeated Replication (BRR), Jackknife, or bootstrap, refit models across replicate samples to obtain valid uncertainty measures [19]. However, while conceptually straightforward, these approaches can be computationally burdensome for high-dimensional Bayesian frameworks like BKMR and raise practical questions about how to combine posterior summaries across replicates. Recent developments attempt to address these limitations. For instance, Das, Bandyopadhyay, and Pati [20] introduced the Survey-adjusted Weighted Likelihood Bootstrap (S-WLB) to provide scalable and efficient inference under complex designs. These emerging approaches demonstrate growing momentum toward design-aware Bayesian inference, yet computational scalability and general integration into mixture-modelling frameworks like BKMR remain open research challenges.

A rapidly developing third route embeds survey features within Bayesian pseudolikelihood or pseudo-posterior frameworks. Early foundations show how weighted likelihoods target population functionals [21,22]. More recent advances provide Bayesian formulations with theoretical guarantees under informative sampling [7,23]. These approaches blend design weights with hierarchical modelling and can incorporate PSU structure, offering a principled path to design-aware Bayesian inference without ad hoc post hoc fixes. Complementary developments in calibrated Bayes and small-area estimation further connect design-based targets to Bayesian computation [24].

Within mixtures analysis, BKMR sits alongside mixtures methods that often handle weights more readily like Weighted Quantile Sum (WQS), quantile g-computation, and Bayesian profile regression [25–29]. However, comparative studies consistently demonstrate that no single mixture-modeling approach performs best across different correlation structures, sparsity levels, and nonlinear interactions, underscoring the importance of honoring sampling design regardless of modeling choice [30,31]. While recent extensions to BKMR such as structure-guided or regularized formulations help stabilize complex exposure response surfaces in high-dimensional settings, they do not address biases introduced by ignored survey features such as sampling weights and clustering [11]. Hence, model choice must be paired with design-aware estimation and appropriate uncertainty quantification. In applied environmental epidemiology, BKMR has been used extensively in cohort and population-based studies to characterize nonlinear mixture effects, with findings often compared against alternative mixture methods to assess robustness [29,31]. These studies provide independent validation of BKMR’s flexibility and practical utility, while also highlighting the importance of aligning modeling approaches with underlying data structures such as complex survey designs.

Building on this foundation, we target practical implementation: instead of modifying BKMR’s likelihood, we resample observations proportional to survey weights while respecting PSUs, so the model sees the weighted empirical distribution and preserves between-cluster variability [32]. This bridges to pseudo-posterior ideas for informative sampling [7] and recent resampling-based solutions for complex surveys [20], aligning with Bayesian perspectives that integrate design-based principles without overhauling the model [33]. We then compare naïve versus weight-aware workflows under informative selection, ICC, and exposure correlation, evaluating bias and coverage against known truth to identify when design awareness matters and when simpler unweighted analyses suffice [3]. In this context, our contribution is to translate these survey-aware ideas into a practical workflow for BKMR, a setting in which such features are not currently available in standard software. Unlike pseudo-posterior approaches that modify the likelihood to directly incorporate survey weights, our framework operates by reweighting the empirical distribution through resampling, allowing design-aware inference without altering the BKMR model structure.

Existing approaches for incorporating survey weights into Bayesian models, such as pseudo-posterior methods, provide a principled framework but are not currently available for BKMR. Therefore, the objective of this study is to develop a computationally feasible and implementation-ready approximation that enables design-aware inference within existing BKMR software.

## 2. Materials and Methods

We developed a structured simulation framework to evaluate naïve and design-aware BKMR under complex survey designs, specifying the data-generating process, survey structure, weight construction, and replication procedure.

### 2.1. Data-Generating Mechanism (Simulation Setup)

We conducted Monte Carlo experiments to evaluate the performance of BKMR for environmental mixture analysis under a two-stage stratified cluster (PSU) survey design, allowing for both non-informative and informative within-cluster sampling. Because the current bkmr R package does not natively accommodate design weights or replicate-weight variance estimation, we developed a design-aware BKMR framework. This approach preserves the BKMR likelihood but modifies the empirical distribution presented to the model by (1) resampling observations proportional to their survey weights and (2) applying a PSU-level bootstrap.

Under established survey-sampling theory, such a weighting plus resampling method approximates estimation under a weighted empirical measure [2,34] and propagates the hierarchical structure of clustering into uncertainty estimation through replication [4]. This interpretation assumes that the survey weights are informative for the target population, that PSU-within-stratum resampling preserves the main dependence structure induced by the design, and that repeated refitting across resamples provides a reasonable approximation to repeated-sampling uncertainty under the complex design. We benchmark this design-aware BKMR against the conventional naïve BKMR, which fits the model directly to the sampled data without accounting for survey design features. The inferential target of this analysis is the superpopulation mean, defined as the expected outcome under the data-generating mechanism rather than a finite-sample population parameter.

### 2.2. Simulation Setup

We simulate a finite population to assess the performance of BKMR under complex survey designs. Each scenario begins with a population of *N_pop_* = 20,000 individuals and M correlated exposures. We considered two simulation settings: a low-dimensional mixture with M = 3 exposures and a higher-dimensional mixture with M = 10 exposures, to evaluate BKMR performance under both simple and more realistic mixture sizes. The exposures are drawn from a mean-zero multivariate normal distribution with an exchangeable correlation structure, where the diagonal elements of the covariance matrix are 1 and the off-diagonal elements equal *ρ* [35]. This approach enables control over exposure collinearity, a common feature of environmental chemical mixtures [5].

Given this exposure structure, the true exposure–response surface is defined by a smooth, nonlinear function incorporating both main effects and interactions:

$$h\left(Z_{1},Z_{2},Z_{3}\right)=1.0Z_{1}Z_{2}+0.5Z_{1}^{2}-0.6Z_{2}^{2}+0.30Z_{3}+0.25Z_{3}^{2}.$$

In simulations with *p* = 10 exposures, the exposure–response function extends the base model for Z_1_–Z_3_ by adding small linear effects for the remaining exposures:

$$h\left(Z_{1},\ldots ,Z_{10}\right)=h\left(Z_{1},Z_{2},Z_{3}\right)+\sum_{k=4}^{10}0.1\;Z_{k}.$$

The outcome for individual *i* within cluster *c* is modeled as:

$$Y=h\left(Z_{1},Z_{2},Z_{3}\right)+u_{c}+\varepsilon ,$$

where $\varepsilon ∼𝒩\left(0,\sigma_{\varepsilon }^{2}\right)$ is random noise, and $u_{c}$ is a cluster-level random effect inducing intraclass correlation (ICC). The ICC is calibrated by setting $Var\left(u_{c}\right)=\sigma_{u}^{2}$ such that

$$ICC=\frac{\sigma_{u}^{2}}{\sigma_{u}^{2}+\sigma_{\varepsilon }^{2}},$$

with $\sigma_{\varepsilon }^{2}=1$. The cluster-level variance was set to $\sigma_{u}^{2}=0\;(ICC=0)$ and $\sigma_{u}^{2}=0.1765\;(ICC=0.15)$ based on the ICC calibration formula. This structure emulates correlation among individuals within primary sampling units (PSUs), reflecting realistic survey data dependencies. The three-exposure setting allows clear visualization and interpretation of nonlinear effects and interactions, while the ten-exposure setting reflects the higher dimensionality and correlation structures commonly encountered in environmental mixture studies. This design allows us to assess how increasing mixture complexity affects estimation, uncertainty, and variable selection under complex survey sampling.

### 2.3. Survey Design and Sampling

The simulated population is divided into *C* = 120 PSUs (clusters), which are assigned cyclically to *H* = 6 strata. Within each stratum, $k_{h}=4\;\mathrm{PSUs}$ are selected without replacement.

We implement two within-PSU sampling regimes:

1. Non-informative sampling (SRS): Within each selected PSU, *m_c_* units are sampled simple-randomly, yielding total sample sizes of *n* ∈ {300, 800, 1200}.
2. Informative sampling (PPS-like): Within PSU *c*, selection probabilities are proportional to a shifted positive function of the first exposure variable $Z_{1}$:
$$p_{ic}\;\propto \;max\left(Z_{1i}-\underset{j\in c}{min}Z_{1j}+10^{-6},0\right),$$

This makes inclusion probabilities depend on exposure. This design is intentionally informative because the inclusion mechanism is linked to variables influencing the outcome *Y* through h(⋅) [36].

Approximate inclusion probabilities are given by:

$$\pi_{i}\approx \frac{k_{h}}{\left|C_{h}\right|}\times Pr\left(i|c\right),$$

where Pr(i∣c) corresponds to either $m_{c}/N_{c}$ for SRS or $min\left(1,m_{c}p_{ic}\right)$ for PPS-like sampling. This setup follows standard two-stage design conventions [2,34].

### 2.4. Construction of Sampling Weights

Base weights are computed as $w_{i}=1/\pi_{i}$. To improve numerical stability, weights are rescaled to have a mean of 1 within the analysis sample. This rescaling does not alter the target estimand, as only relative weights influence the resampling probabilities. The inferential target is the superpopulation mean, and the resampling procedure is intended to approximate repeated-sampling variability under the complex survey design rather than to recover a formal Bayesian posterior. In the context of BKMR, which relies on kernel-based distance calculations, extreme weight values can disproportionately influence local smoothing and lead to instability; rescaling mitigates this while preserving the underlying weighted empirical distribution. This adjustment maintains relative weighting and effective sample size while preventing instability in BKMR estimation. No trimming is applied in the primary analysis. This preserves the original sampling design and allows the design-aware framework to be evaluated under the observed weight distribution. Although trimming may improve numerical stability in the presence of extreme weights, systematic evaluation of trimming thresholds was beyond the scope of the current study and is an important direction for future work [2].

#### Estimands and Targets of Inference

The primary estimand mirrors the overall mixture effect in BKMR studies [5]:

$$\Delta^{*}=h\left(q_{0.75}\right)-h\left(q_{0.25}\right),$$

where $q_{p}=\left(q_{p}\left(Z_{1}\right),q_{p}\left(Z_{2}\right),q_{p}\left(Z_{3}\right)\right)$ denotes the *p*-th percentile of each exposure’s marginal distribution. Because h(⋅) is known in simulation, $\Delta^{*}$ represents the true effect. Secondary targets include univariate and bivariate exposure–response functions, as well as pairwise interaction slices. In the design-aware workflow, the goal is to recover this target at the population level rather than the sample level. Accordingly, the weighted resampling procedure is intended to re-express the observed sample as an approximation to the population-weighted empirical distribution seen by BKMR.

### 2.5. Analysis Workflows

Two BKMR workflows are compared:

- Naïve BKMR (unweighted): BKMR is fitted directly to the survey sample (*Y, Z*) without incorporating survey design features. This reflects standard use when only analytic weights are unavailable or ignored.
- Design aware BKMR (weight-proportional resampling): Because BKMR lacks built-in options for weights or clusters, we emulate design-weighted learning through a two-stage resampling approach [4,37]:
  1. PSU Stage: Resample PSUs with replacement using probabilities proportional to the PSU’s mean weight ${\bar{w}}_{c}$.
  2. Unit Stage: Within each selected PSU, resample individuals with replacement using probabilities proportional to $w_{i}$.

This approximates importance-weighted learning while maintaining between-PSU variability [34]. Optional replicate-weight or PSU bootstrapping is used to obtain design-consistent uncertainty estimates [4]. Because uncertainty is generated through repeated resampling rather than a single posterior distribution, the resulting interval estimates should be interpreted as design-consistent, replication-based uncertainty intervals rather than pure Bayesian posterior intervals.

#### Algorithmic Implementation Details

For each design-aware analysis, PSU resampling is conducted independently within each stratum. Let *H_s_* denote the number of PSUs in stratum s. We sample *H_s_* PSUs with replacement, with selection probabilities proportional to the sum of survey weights within each PSU. Conditional on the selected PSUs, individuals are resampled with replacement within each PSU using probabilities proportional to their sampling weights. This two-stage procedure preserves the stratified cluster structure while approximating a weighted empirical distribution. For uncertainty estimation, this procedure is repeated across B = 50 bootstrap replicates, and BKMR is refit on each resampled dataset.

### 2.6. Tuning, Computation, and Convergence

BKMR models were fitted using the default Gaussian radial basis function (RBF) kernel and priors, with variable selection enabled (varsel = TRUE) to allow stochastic inclusion of exposures during MCMC sampling [5]. Each fit runs 4000 iterations, with pilot analyses confirming stability of posterior summaries. Convergence is assessed through trace plots of the overall effect, kernel scale parameters, and regression variance. The potential scale reduction factor $\hat{R}$ near 1 indicates convergence. Computation is parallelized across Monte Carlo replicates using independent random seeds.

For the main analysis, BKMR models were run for 4000 iterations with a burn-in of 2000 and thinning of 2, yielding 1000 posterior samples. For bootstrap replicates, shorter chains were used (2500 iterations, burn-in = 1000, thinning = 2) to balance computational cost and stability. These settings were selected based on pilot runs to ensure stable posterior summaries while maintaining feasible runtime. We note that formal MCMC convergence diagnostics (e.g., ESS and Geweke statistics) were not systematically evaluated across all resampling replicates and remain an area for future work.

Accordingly, posterior inclusion probabilities and other MCMC-based summaries should be interpreted with caution, particularly in the resampling-based analyses.

#### 2.6.1. Posterior Inclusion Probabilities (PIPs) in Simulations and Real Data

Posterior inclusion probabilities in BKMR reflect contribution to the kernel representation of the exposure-response surface and should not be interpreted as formal hypothesis tests for individual exposures. In settings with a small number of exposures or strong signal, PIPs may concentrate near one due to limited competition among variables, and should therefore be interpreted cautiously as indicators of inclusion rather than measures of relative importance or effect size.

In the survey-aware framework, PIPs are also influenced by resampling variability across bootstrap replicates. Although we summarize PIPs as posterior means, variability across resamples provides an implicit measure of selection stability; exposures consistently selected across resamples are interpreted as more robust, whereas variability in inclusion suggests sensitivity to sampling variability. Formal quantification of PIP variability across replicates was not performed and remains an important direction for future work.

To evaluate how complex survey design affects variable selection in BKMR, we enabled BKMR variable selection (varsel = TRUE) and summarized exposure importance using PIPs. Let $\delta_{j}^{\left(t\right)}\in \{0,1\}$ denote the inclusion indicator for exposure *j* at MCMC iteration *t* after burn-in. The variable-level PIP was estimated as the posterior probability that an exposure was included in the BKMR kernel function:

$${PIP}_{j}=P\left(\delta_{j}=1\;|\;\text{data}\right)\;\approx \;\frac{1}{T}\sum_{t=1}^{T}\delta_{j}^{\left(t\right)},$$

where *T* is the number of retained post–burn-in iterations. PIPs were computed for both the naïve BKMR fit and the survey-aware fit obtained via weight-proportional resampling, using PSU bootstrap resampling when clustering was present.

We evaluated PIPs under two exposure dimensions: a low-dimensional setting (*p* = 3) and a higher-dimensional setting (*p* = 10). In the *p* = 3 setting, variable selection is expected to be less informative because there are only three candidate exposures and limited competition for inclusion; as a result, PIPs may concentrate near one even when effects are modest. In the *p* = 10 setting, selection is more discriminating because exposures compete to explain the outcome, allowing PIPs to better separate strong-signal exposures from weaker ones.

Because the simulation does not contain natural chemical classes, we defined groups as singleton sets (one exposure per group) for consistency with the BKMR implementation. Under this definition, group-level PIPs reduce to the corresponding variable-level PIPs (i.e., ${PIP}_{g,\text{any}}={PIP}_{j}$ when g={j}). Conditional PIPs were also computed as a co-selection diagnostic,

$${cPIP}_{i|j}=P\left(\delta_{i}=1\;|\;\delta_{j}=1\right),$$

but they become less informative when inclusion is nearly saturated (e.g., when many $\delta_{j}^{\left(t\right)}$ values are equal to 1), in which case ${cPIP}_{i|j}$ can approximate ${PIP}_{i}$. Therefore, we emphasize variable-level PIPs as the primary selection summary in the simulation study. We note that when inclusion probabilities are near one across many exposures, conditional and group-level PIPs provide limited additional discrimination, and interpretation should rely more heavily on complementary summaries such as exposure–response functions and risk contrasts.

In the real-data analysis, PIPs were computed using the same definition, but group and conditional PIPs are substantively meaningful because exposures belong to predefined chemical classes. Group-level PIPs were estimated directly from the MCMC inclusion indicators. Specifically, for each chemical group *g*, the group PIP was defined as the posterior probability that at least one exposure within the group was included in the BKMR kernel,

$${\mathrm{GroupPIP}}_{g}=\frac{1}{T}\sum_{t=1}^{T}I\left(\sum_{j\in g}\delta_{jt}>0\right)$$

where $\delta_{jt}$ denotes the inclusion indicator for exposure *j* at MCMC iteration *t*. This definition properly accounts for dependence among exposures and inclusion indicators.

Along with the group mean and maximum PIP as descriptive summaries, to characterize co-selection patterns among exposures in the mixture, we also computed conditional PIPs,

$${cPIP}_{i|j}=P\left(\delta_{i}=1\;|\;\delta_{j}=1\right),$$

estimated from the MCMC inclusion indicators as the proportion of iterations in which exposure *i* was included among those iterations where exposure *j* was included. We report PIP results for both naïve and survey-aware analyses to assess how complex survey structure influences variable selection and co-selection patterns in population-based mixture analyses.

#### 2.6.2. Combining Results Across Resamples and Imputations

For each BKMR fit (either naïve or design-aware), summary quantities such as univariate exposure–response functions, bivariate surfaces, and overall mixture effects were computed using standard BKMR post-processing functions. For the design-aware workflow, these summaries were computed separately within each bootstrap replicate. Scalar summaries (e.g., overall mixture effect contrasts) were aggregated across replicates using empirical means, and uncertainty was quantified using percentile-based bootstrap intervals (2.5th and 97.5th percentiles).

When multiple imputation was used, BKMR was fit separately to each imputed dataset. Scalar estimates were combined using Rubin’s rules, while functional summaries (e.g., exposure–response curves) were averaged pointwise across imputations.

#### 2.6.3. Computation

The design-aware workflow increases computational cost relative to a single BKMR fit because the model must be refit across bootstrap replicates. In our implementation, this corresponds to approximately 50 BKMR refits for the design-aware workflow relative to one fit for naïve BKMR, because B = 50 bootstrap replicates were used. To address this, all resampling-based analyses were parallelized across cores, with each replicate evaluated independently. As a result, wall-clock time was reduced through parallel processing, although total computational cost increased with the number of resamples.

### 2.7. Performance Metrics

To evaluate estimator performance, we assessed operating characteristics across *R* = 100 Monte Carlo replicates for each simulation scenario. All metrics were computed for the population-level overall mixture effect ∆, defined as the contrast in the outcome when all exposures jointly increase from their 25th to 75th percentiles.

Bias quantifies systematic deviation of the estimator from the true population value and is defined as

$$\text{Bias}=\frac{1}{R}\sum_{r=1}^{R}\left({\overset{ˆ}{\Delta }}_{r}-\Delta^{*}\right),$$

where ${\overset{ˆ}{\Delta }}_{r}$ is the estimate from replicate r and $\Delta^{*}$ is the true value under the data- generating process.

Interval width measures estimator precision and it was summarized using the average width of the 95% interval across Monte Carlo replicates. Uncertainty calibration was evaluated through empirical coverage, defined as the proportion of replicates in which the 95% uncertainty interval contained the true population effect Δ*. Although BKMR is a Bayesian model, coverage was assessed using frequentist criteria to examine the repeated-sampling performance of posterior uncertainty. Overall estimation accuracy was further summarized using the root mean square error (RMSE), which combines both bias and variability and is defined as

$$RMSE=\sqrt{𝔼\left[{\left(\overset{ˆ}{\Delta }-\Delta^{*}\right)}^{2}\right]}.$$

Finally, we summarize calibration by comparing empirical coverage to the nominal 95% level, with under-coverage indicating over-confident inference and over-coverage indicating conservative uncertainty. These metrics together allow direct comparison of naïve and design-aware BKMR in terms of bias, precision, and uncertainty calibration under complex survey sampling.

It is important to distinguish two sources of uncertainty. For a single BKMR fit, uncertainty is summarized using posterior credible intervals derived from the Bayesian model. In contrast, for the design-aware workflow, uncertainty reflects both model uncertainty and sampling variability and is summarized using replication-based (bootstrap) intervals across resampled datasets. These intervals should therefore be interpreted as design-consistent uncertainty intervals rather than posterior credible intervals from a single model fit.

### 2.8. Experimental Design

We used a full factorial experimental design to evaluate the performance of naïve and survey-aware BKMR across a range of realistic sampling conditions. Four key factors were varied: sample size (*n* ∈ {300, 800, 1200}), exposure correlation (*ρ* ∈ {0, 0.8}), clustering strength as measured by the intraclass correlation coefficient (ICC ∈ {0, 0.15}), and sampling informativeness (non-informative simple random sampling versus informative PPS-like sampling). Each unique combination of these factors defined a simulation scenario.

For each scenario, we generated *R* = 100 independent Monte Carlo replicates to assess estimator performance under repeated sampling. Random number generation was controlled using L’Ecuyer–CMRG streams to ensure reproducibility across parallel runs [38]. Performance metrics including bias, interval width, coverage, and RMSE were computed by aggregating results across replicates within each scenario.

All simulations and analyses were conducted in R version 4.3. BKMR models were fit using the bkmr package, with multivariate normal exposures generated using MASS. Data manipulation and visualization relied on dplyr, tidyr, ggplot2, and scales, while computational efficiency was achieved through parallelization using future.apply. All code used to generate the results, including preprocessing, model fitting, resampling, and plotting, is provided in the accompanying GitHub repository.

### 2.9. Inferential Framework and Interpretation

The proposed approach is a design-based approximation rather than a fully model-based Bayesian method. Specifically, survey weights are incorporated through PSU-level resampling, and uncertainty is quantified across resampled datasets. As a result, the intervals obtained from the design-aware workflow should be interpreted as replication-based uncertainty intervals rather than posterior credible intervals.

This distinction is important because the method does not define an explicit weighted likelihood or posterior distribution. Instead, it approximates the weighted empirical distribution induced by the sampling design. Consequently, the approach is intended to provide a practical solution for incorporating complex survey features into BKMR, rather than a fully theoretically grounded Bayesian framework.

## 3. Results

### 3.1. Bias of the Estimated Overall Mixture Effect

**Figure 1.**
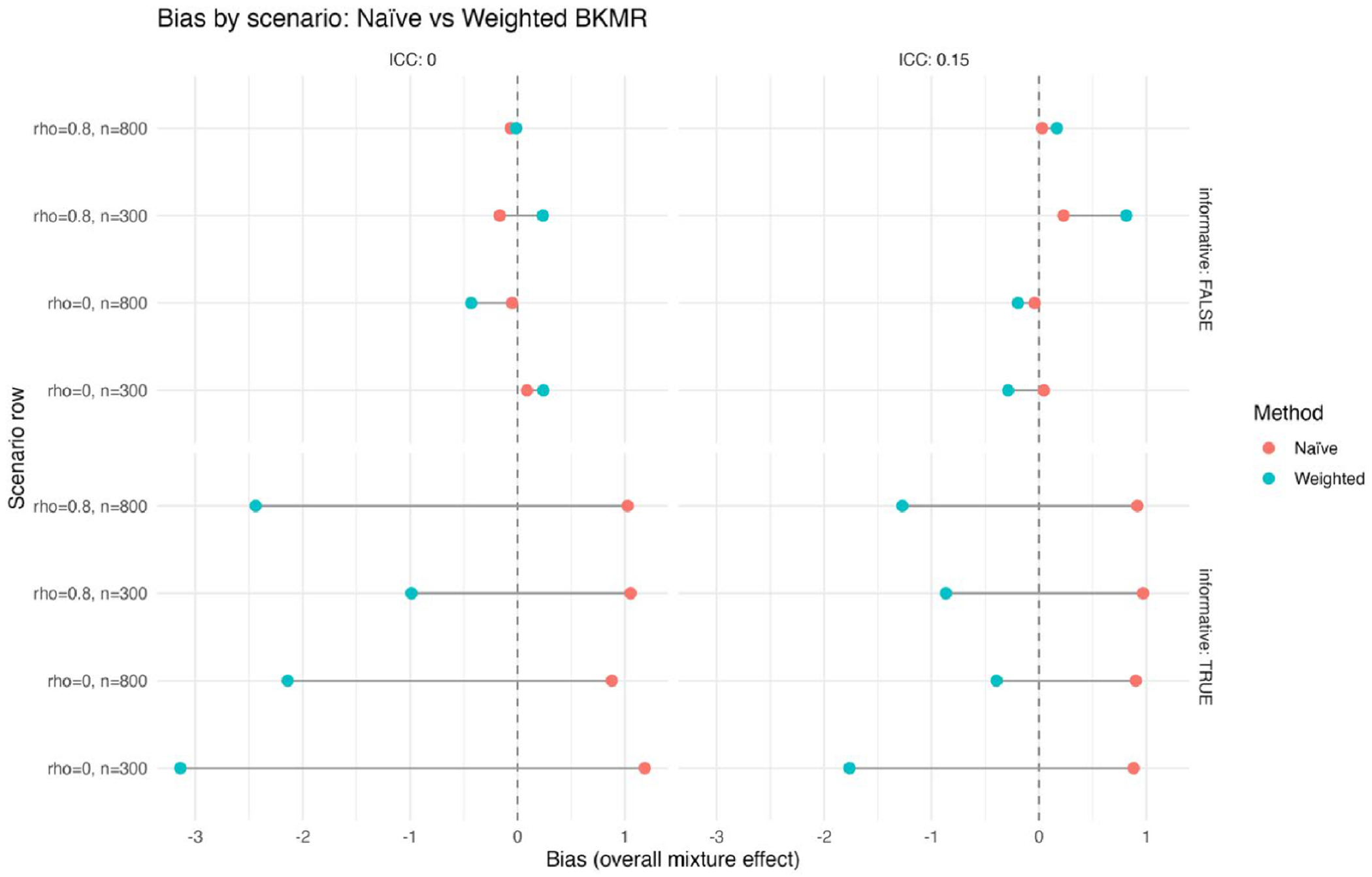
Bias of the estimated overall mixture effect by design scenario, comparing naive vs. weighted BKMR.

Figure 1 shows bias in estimated overall mixture effects by design scenario for naïve versus weighted BKMR. Each point shows the average bias from simulation runs comparing naïve BKMR and design-aware weighted BKMR. Scenarios vary by exposure correlation (ρ = 0 or 0.8), sample size (*n* = 300 or 800), and (ICC = 0 or 0.15). The top panels show results under non-informative sampling, where selection is unrelated to exposure; both methods produce near-zero bias, especially with larger *n*. The bottom panels represent informative sampling, where exposure affects inclusion probability; naïve BKMR tends to overestimate effects (points shifted right of zero), while the weighted approach reduces bias, sometimes slightly overcorrecting with small samples or high exposure correlation. ICC alone does not cause bias but increases variability when sampling is informative. This shows that weighting aligns estimates closer to the true population effect, especially under unequal-probability sampling.

#### 3.1.1. Posterior Inclusion Probabilities (PIPs)

Tables 1 and 2 report PIPs from BKMR under two simulation scenarios, comparing naïve and survey-weighted analyses as the number of exposures increases.

Table 1 shows results for a low-dimensional mixture with three exposures (Z_1_–Z_3_). In this setting, all exposures and their corresponding groups have PIPs equal to one under both naïve and survey-weighted BKMR, indicating clear and consistent identification of all mixture components as relevant to the outcome. This result reflects the strong signal in the data-generating process and the limited competition among exposures. When inclusion probabilities are concentrated near one, exposure-level and group-level PIPs coincide, since inclusion is nearly certain across posterior samples. In this setting, PIPs close to one are expected and do not provide meaningful differentiation among exposures but instead indicate that all components of the mixture contribute to the outcome.

The similarity between naïve and weighted PIPs in this setting further indicates that, when signal strength is high and mixture complexity is low, accounting for survey design mainly affects uncertainty estimation rather than variable selection. Under these conditions, both approaches recover the correct set of relevant exposures.

Table 2 presents result for a higher-dimensional mixture with ten exposures (Z_1_–Z_10_), where variable selection is more challenging. Although the first three exposures retain PIPs equal to one under both models, several additional exposures show reduced PIPs under the naïve analysis, reflecting increased competition among mixture components and weaker posterior support for smaller effects as dimensionality increases.

In contrast, the survey-weighted BKMR assigns higher PIPs to many of these exposures and their corresponding groups, indicating that accounting for unequal selection probabilities and clustering helps preserve evidence for relevant mixture components. These differences arise from reweighting the empirical exposure distribution to better reflect the population structure, rather than from changes to the BKMR likelihood itself.

These results show that mixture dimensionality strongly influences BKMR variable selection and that ignoring complex survey design can lead to attenuation of PIPs in higher-dimensional settings. This pattern is consistent with the real-data findings in Table 2, reinforcing the importance of design-aware BKMR for analyzing complex exposure mixtures in population-based surveys.

Posterior inclusion probabilities (PIPs) reflect model-based variable selection under the specified framework and may be influenced by resampling variability. Higher PIPs under the design-aware approach should therefore not be interpreted as definitive evidence of stronger causal importance, but rather as indicators of stability within the resampling framework.

#### 3.1.2. Univariate Exposure–Response Functions

**Figure 2.**
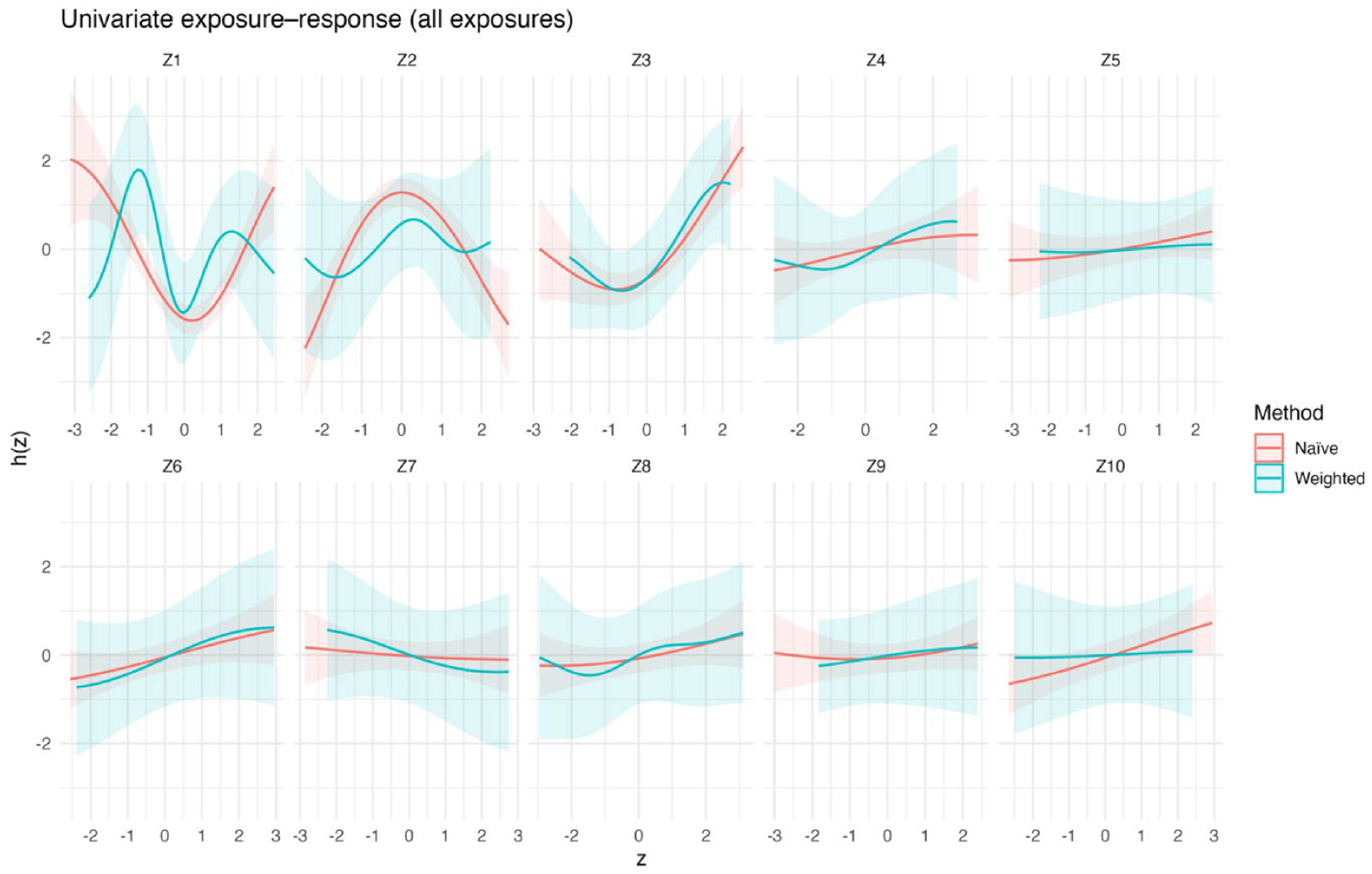
Univariate exposure–response functions (Z1–Z10) comparing naïve and survey-weighted BKMR.

Figure 2 presents the estimated univariate exposure–response functions for exposures Z1–Z10, comparing naïve BKMR with survey-weighted BKMR that accounts for the complex sampling design. For each exposure, the curves depict the posterior mean of the exposure–response function, holding all other exposures fixed at their median values. The shaded bands represent 95% intervals; for naïve BKMR these are posterior credible intervals, whereas for survey-weighted BKMR they represent replication-based uncertainty intervals.

Across the ten exposures, the estimated univariate exposure–response functions show varied patterns. For some exposures, including Z1–Z3, both naïve and survey-weighted BKMR indicate nonlinear relationships with the outcome, although the shape and strength of these associations differ between the two methods, especially at the lower and upper ends of the exposure range. In general, the survey-weighted estimates tend to have wider uncertainty intervals, reflecting additional uncertainty from sampling weights and clustering, while changes in smoothness differ across exposures. For the remaining exposures (Z4–Z10), the estimated associations are generally weak and close to zero over most of the exposure range, with substantial overlap between naïve and weighted credible intervals. In these cases, differences between methods are mainly due to increased uncertainty rather than consistent changes in effect size, with the weighted model often showing greater variability near the edges of the standardized exposure range. Supplementary Figure S1 shows the corresponding univariate exposure–response functions for the three-exposure setting.

#### 3.1.3. Bivariate Exposure–Response Functions

**Figure 3.**
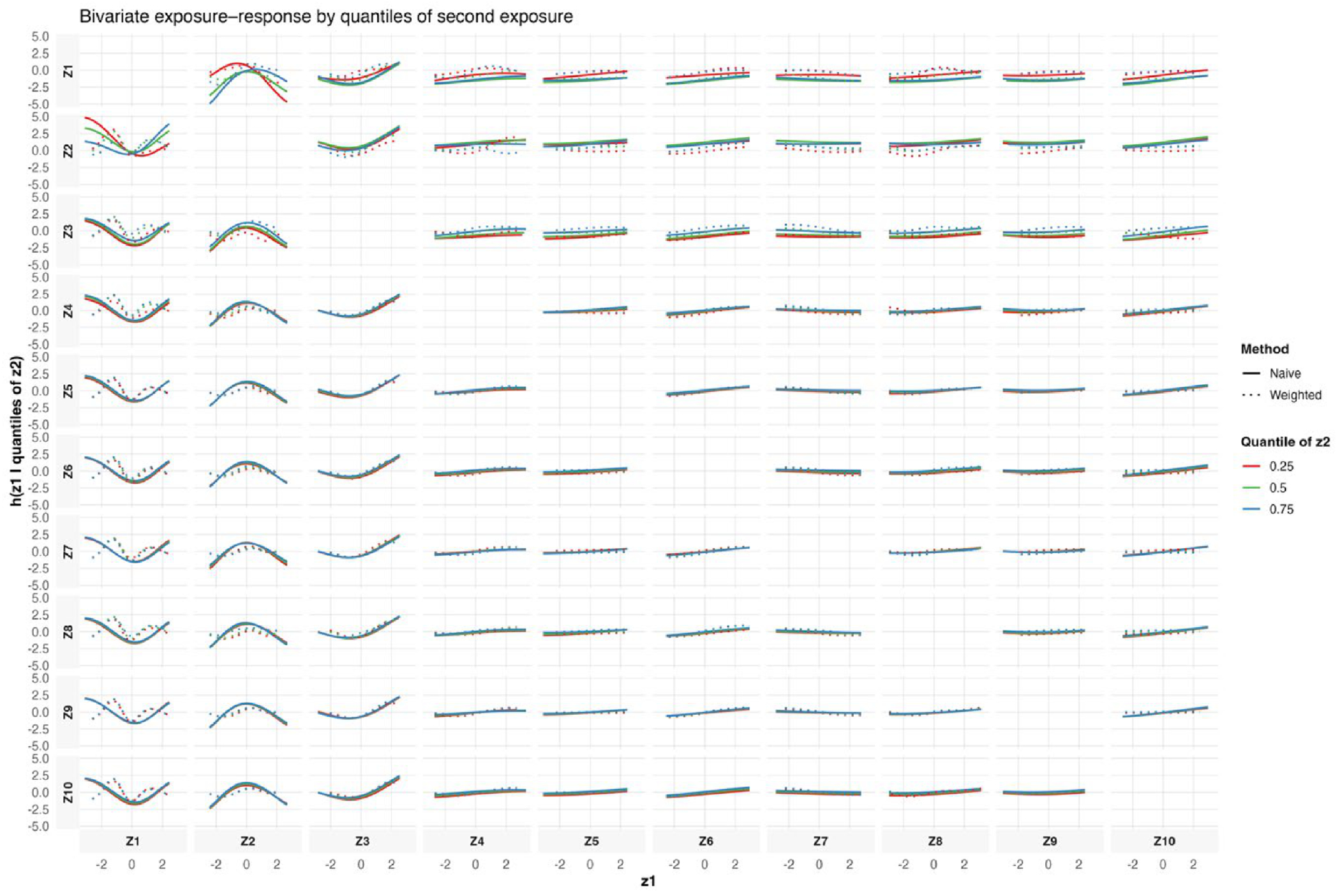
Bivariate exposure–response functions from naïve and survey-weighted BKMR.

Figure 3 presents bivariate exposure–response functions estimated using BKMR, stratified by quantiles of a second exposure. Each panel shows the relationship between a focal exposure (*x*-axis) and the outcome, conditional on the second exposure fixed at its 25th, 50th, or 75th percentile, while holding all remaining mixture components at their median values. Results are shown for both the naïve model and survey-weighted BKMR model. For exposure pairs involving Z1–Z3, the estimated exposure–response relationships vary across conditioning quantiles, indicating evidence of nonlinear interaction effects within this subset of the mixture. These interactions are more pronounced in the naïve analysis, with greater curvature and wider separation between quantile-specific curves. After accounting for the complex survey design, the corresponding weighted estimates exhibit smoother response patterns and increased uncertainty, suggesting partial attenuation of interaction effects once population weights and clustering are incorporated. For exposure pairs involving Z4–Z10, conditional associations are generally weak and closer to linear across quantiles, with minimal separation between curves. This pattern suggests limited interaction effects for these exposure combinations and indicates that the inferred associations are relatively stable across different levels of the conditioning exposure. Supplementary Figure S2, shows these relationships when there are three exposures.

#### 3.1.4. Single-Variable Risk Summaries

**Figure 4.**
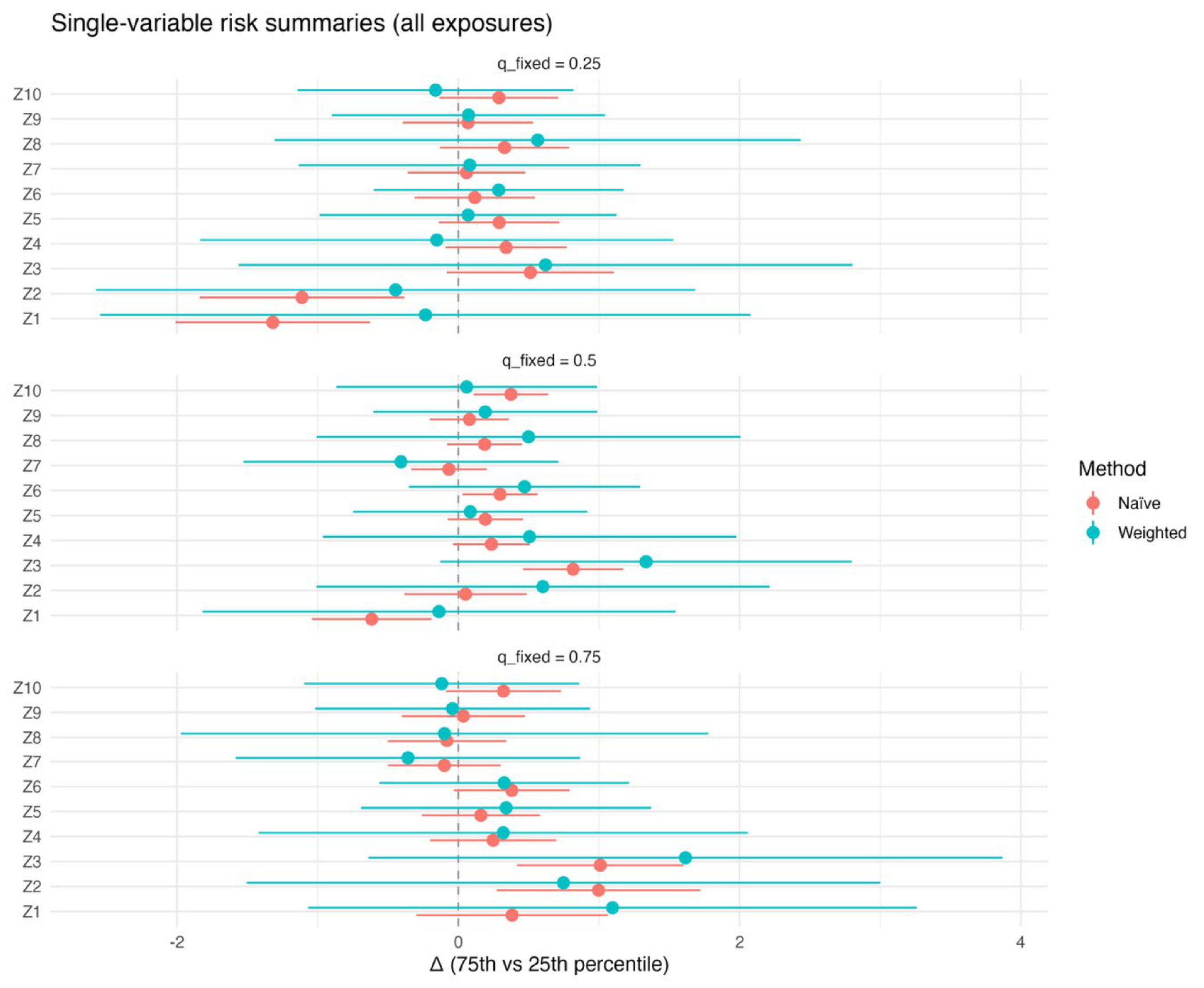
Single-variable effects with and without survey weighting.

Figure 4 presents the single-variable risk summaries for all mixture components (Z1–Z10), comparing naïve and survey-weighted approaches. For each exposure, the plotted point estimates represent the estimated change in the outcome when the exposure is increased from the 25th to the 75th percentile, while all other mixture components are fixed at a specified quantile. Results are shown for three conditioning scenarios, with the remaining exposures fixed at the 25th, 50th, and 75th percentiles.

Across conditioning levels, exposures Z1–Z3 exhibit the largest estimated risk differences under both modeling approaches, although the survey-weighted estimates tend to be attenuated and accompanied by wider uncertainty intervals. For exposures Z4–Z10, estimated risk differences are generally modest and often include the null, with limited variation across conditioning quantiles. Differences between naïve and weighted estimates are more evident for exposures with stronger underlying associations, reflecting the influence of incorporating complex survey design on marginal risk summaries. Supplementary Figure S3 shows these relationships when there are three exposures.

#### 3.1.5. Overall BKMR Mixture Effect

**Figure 5.**
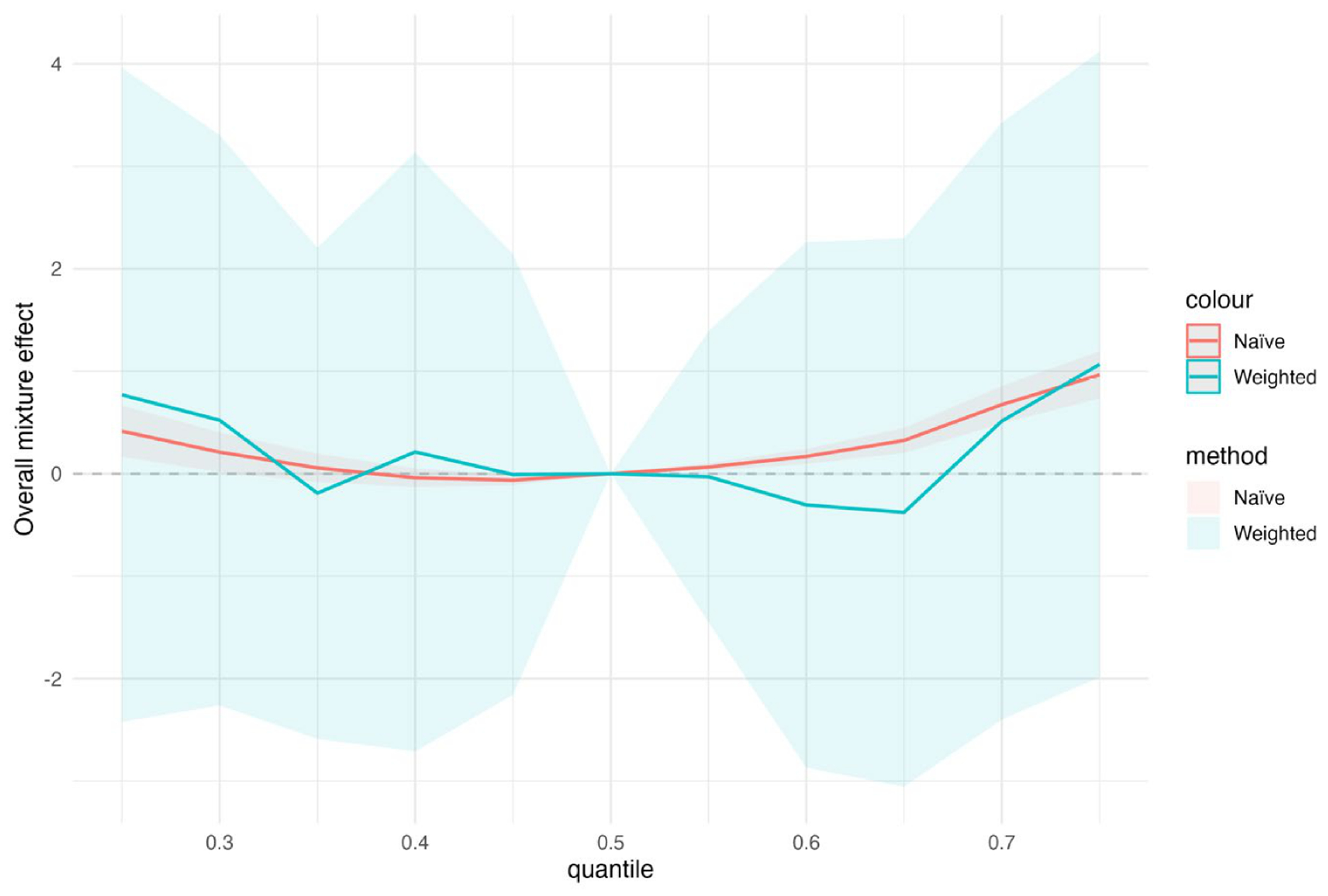
Overall BKMR mixture effect by exposure quantile.

Figure 5 presents the overall BKMR mixture effect by exposure quantile with and without survey weighting. Figure 5 displays the estimated overall BKMR mixture effect across exposure quantiles for naïve and survey-weighted analyses. Each curve represents the estimated change in the outcome when all exposures are jointly set to a given quantile, relative to the median exposure level (q = 0.50). Shaded regions denote 95% uncertainty intervals, and the dashed horizontal line indicates no effect. Across quantiles, the survey-weighted analysis produces estimates that remain close to zero and are accompanied by substantially wider uncertainty intervals, reflecting increased variability after accounting for unequal selection probabilities and clustering. These intervals frequently overlap zero, indicating limited evidence of a strong cumulative mixture effect once the complex survey design is incorporated. In contrast, the naïve analysis shows a more pronounced upward trend at higher exposure quantiles, with comparatively narrower uncertainty intervals, suggesting greater apparent precision. This pattern is consistent with inflated overall mixture effects when the complex survey design is ignored. Supplementary Figure S4 presents the corresponding results for the three-exposure scenario.

Unless otherwise stated, intervals from the weighted analysis represent replication-based uncertainty intervals, whereas intervals from single BKMR fits correspond to posterior credible intervals.

### 3.2. Application to Nationally Representative Survey Data

To complement the simulation study and demonstrate the practical implications of accounting for complex survey design in BKMR, we applied the proposed design-aware workflow to data from the 2013–2014 cycle of the National Health and Nutrition Examination Survey (NHANES). This application serves two purposes. First, it illustrates how the methodological differences identified in simulation translate to a real, population-based setting. Second, it allows direct comparison of naïve and survey-weighted BKMR in a context where unequal selection probabilities and clustering are intrinsic to the data-generating process. In the real-data application, the design-aware workflow was implemented using 50 PSU bootstrap replicates, with BKMR refitted on each resampled dataset. Summary estimates and uncertainty intervals were obtained by aggregating results across these replicates, consistent with the simulation framework.

For a single BKMR fit, uncertainty is summarized using posterior credible intervals derived from the Bayesian model. However, for the design-aware weighted workflow, uncertainty is generated through repeated PSU-within-stratum resampling prior to model refitting. As a result, the resulting intervals are not interpreted as posterior credible intervals from a single Bayesian model, but rather as replication-based (bootstrap-style) intervals that reflect repeated-sampling variability under the complex survey design. This distinction follows established survey inference approaches and aligns with discussions of Bayesian inference under informative sampling.

#### 3.2.1. Study Population and Exposure Characterization

The analytic sample included 4345 adult participants from the 2013–2014 cycle of the NHANES. The outcome of interest was total cholesterol (TotalCholesterol), a well-established indicator of cardiovascular risk. We examined a high-dimensional mixture of environmental chemicals encompassing major exposure classes commonly studied in environmental health, including 13 metals, 9 per- and polyfluoroalkyl substances (PFAS), and 11 phthalates and plasticizers. Metal concentrations were measured in micrograms per liter (μg/L), whereas PFAS and phthalate metabolites were measured in nanograms per milliliter (ng/mL). All models adjusted for a consistent set of individual-level covariates, including age, ethnicity, income, gender, body mass index (BMI), smoking status, and alcohol use.

Missing data were addressed using multiple imputation implemented with the Amelia package in R, which applies an expectation–maximization algorithm with bootstrapping. We generated 10 imputed datasets. In principle, BKMR models should be fit separately within each imputed dataset, with scalar estimates combined using Rubin’s rules and functional summaries averaged pointwise across imputations. Due to the substantial computational burden of BKMR under the resampling framework, we interpret the NHANES results as a demonstrative application rather than a fully inferential analysis [39,40]. NHANES employs a complex, multistage probability sampling design; therefore, to obtain population-representative inference, we incorporated the PSU and stratum variables, along with the 2-year Mobile Examination Center (MEC) examination weight, which adjusts for unequal selection probabilities and nonresponse and ensures nationally representative inference for participants who completed the NHANES physical examination and laboratory assessments. Naïve BKMR analyses ignored the survey design, whereas the design-aware approach incorporated it through PSU bootstrap resampling within strata, with resampling probabilities informed by the survey weights to approximate population-representative inference.

Table 3 reports unweighted descriptive statistics for each analyte, including sample size, arithmetic mean, and standard deviation. These summaries characterize the exposure landscape underlying the mixture analysis and highlight the substantial heterogeneity and right-skew typical of biomonitoring data. As expected for NHANES, exposure distributions vary markedly across chemical classes and individual analytes, underscoring the need for flexible, nonlinear modeling approaches such as BKMR.

#### 3.2.2. Variable Selection Under Naïve and Design-Aware BKMR

We next fit BKMR models to the NHANES data using both the naïve (unweighted) approach and the proposed design-aware workflow that incorporates survey weights and clustering through probability-proportional resampling with PSU preservation. Variable selection was enabled in all models, and PIPs were used to summarize the relative importance of individual exposures and exposure groups.

Table 4 reports exposure-level (conditional) and group-level PIPs from the NHANES analysis under naïve and design-aware BKMR. Conditional PIPs measure the marginal importance of each exposure within the mixture, while group-level PIPs summarize the probability that at least one exposure within a chemical class contributes to the outcome.

Across all three chemical classes, metals, PFAS, and phthalates, group-level PIPs equal one under both modeling approaches, indicating strong and consistent evidence that each exposure class is jointly associated with the outcome. At the exposure level, conditional PIPs are generally high under both naïve and weighted analyses, suggesting robust identification of key mixture components.

Because group-level PIPs are defined as the probability that at least one exposure within a group is included, they can approach one when multiple correlated exposures contribute to the outcome. In this context, group PIPs primarily indicate the presence of signal within a chemical class rather than the strength or distribution of effects across individual exposures. Accounting for the complex survey design primarily affects the relative strength of evidence at the individual-exposure level. Compared with the naïve analysis, the design-aware BKMR yields modest shifts in conditional PIPs for selected analytes, with some exposures exhibiting attenuated inclusion probabilities after weighting. These changes indicate that incorporating unequal selection probabilities and clustering can influence exposure prioritization within the mixture, even when overall conclusions regarding the relevance of chemical classes remain unchanged.

**Figure 6.**
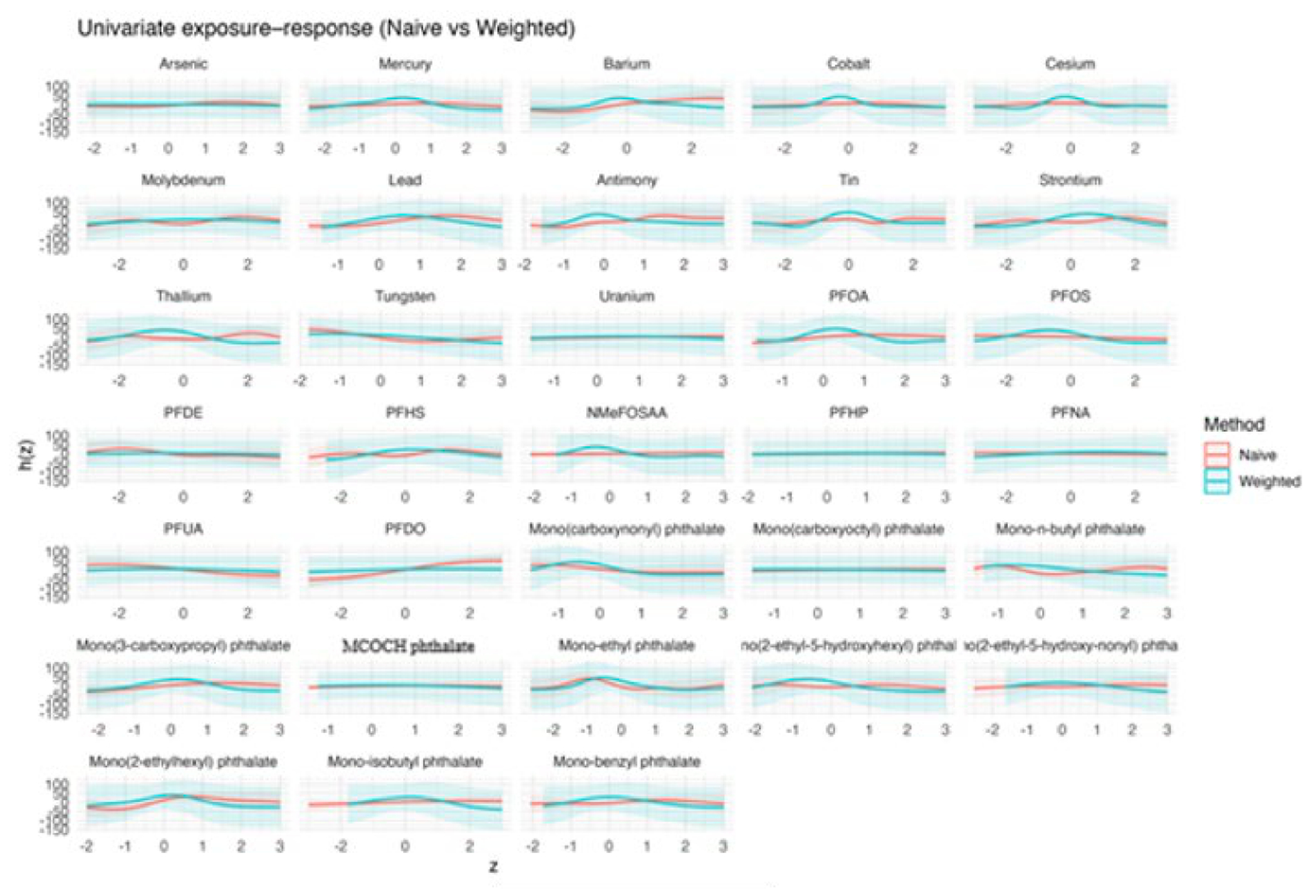
Univariate exposure–response functions from BKMR comparing naïve and survey-weighted analyses.

Figure 6 shows univariate exposure–response functions estimated for each individual exposure in the mixture. Each panel corresponds to a single exposure. For all panels, the remaining exposures in the mixture are held fixed at their median values. Red curves represent estimates from the naïve BKMR analysis that ignores the complex survey design, while teal curves represent estimates from the design-aware analysis based on PSU bootstrap resampling. Solid lines denote posterior mean estimates. Shaded bands indicate 95% intervals; for naïve BKMR these are posterior credible intervals, whereas for survey-weighted BKMR they are replication-based uncertainty intervals. Across many exposures (e.g., several PFAS compounds and selected metals), the naïve and weighted curves exhibit similar overall shapes, suggesting broadly consistent qualitative associations with the outcome. However, notable differences emerge for specific chemicals. For example, some metals display more pronounced curvature in the naïve analysis that is partially attenuated under the weighted analysis, while several phthalate metabolites show wider uncertainty intervals after weighting, reflecting increased uncertainty once population weights and clustering are incorporated. In a few cases, the location of the peak or trough of the response curve shifts modestly between the naïve and weighted analyses, indicating sensitivity of the inferred dose–response relationship to survey design adjustment.

**Figure 7.**
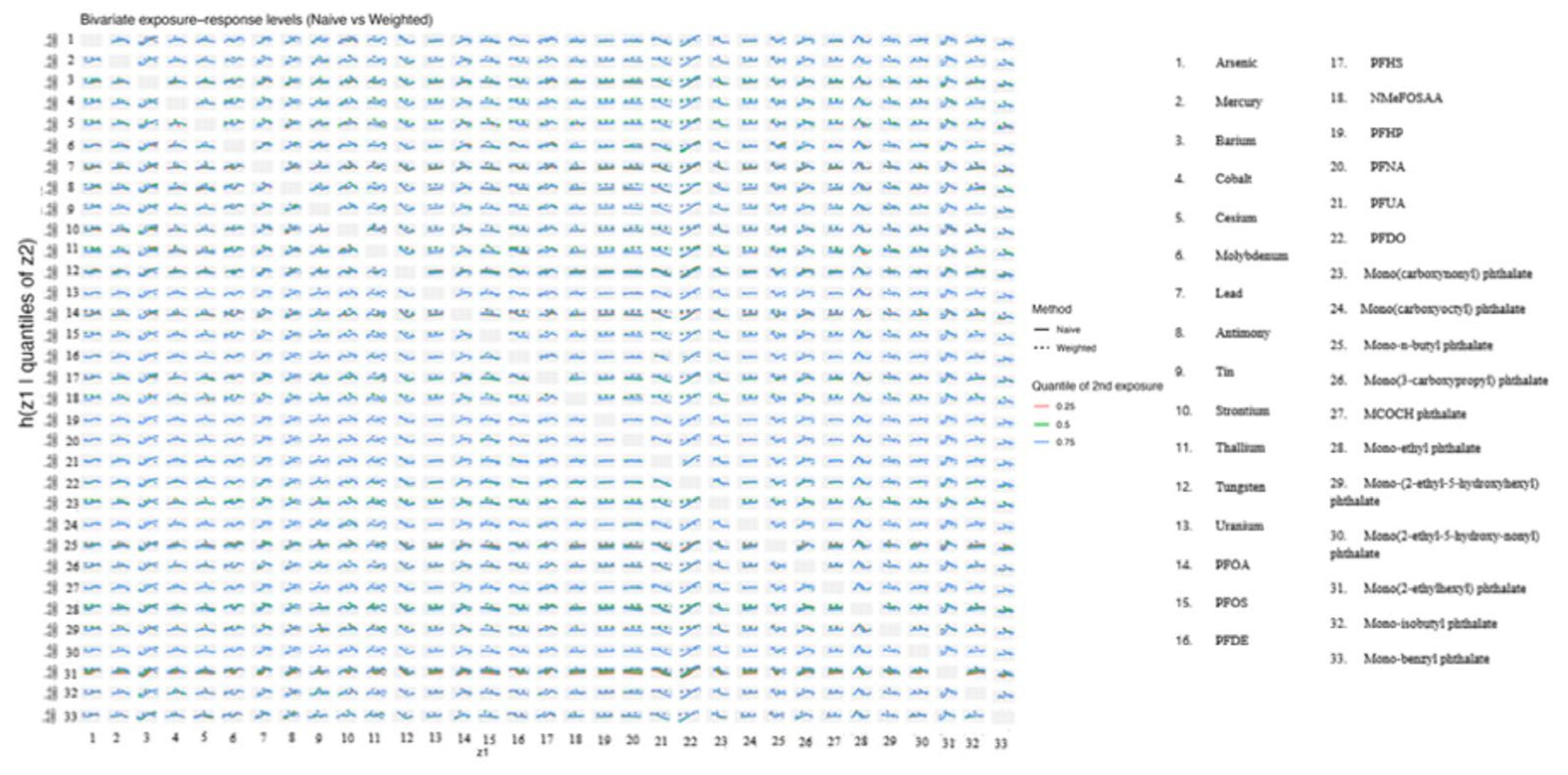
Bivariate exposure–response functions from BKMR comparing naïve and survey-weighted analyses.

Figure 7 shows bivariate exposure–response functions from BKMR for all pairwise combinations of exposures in the mixture. Each panel displays the estimated response as a function of a primary exposure, conditional on a second exposure fixed at its 25th, 50th, or 75th percentile. Within each panel, solid lines represent estimates from the naïve BKMR analysis, while dashed lines represent estimates from the survey-weighted analysis. Colors indicate the quantile level of the second exposure. Differences in curve shape or separation across quantiles reflect potential nonlinear interactions or effect modification between exposure pairs. For many exposure combinations, naïve and weighted curves show similar patterns across quantiles, suggesting broadly consistent interaction structures. However, for some pairs, the naïve analysis exhibits stronger curvature or wider separation between quantile-specific curves, whereas the weighted analysis yields flatter or more closely spaced curves.

**Figure 8.**
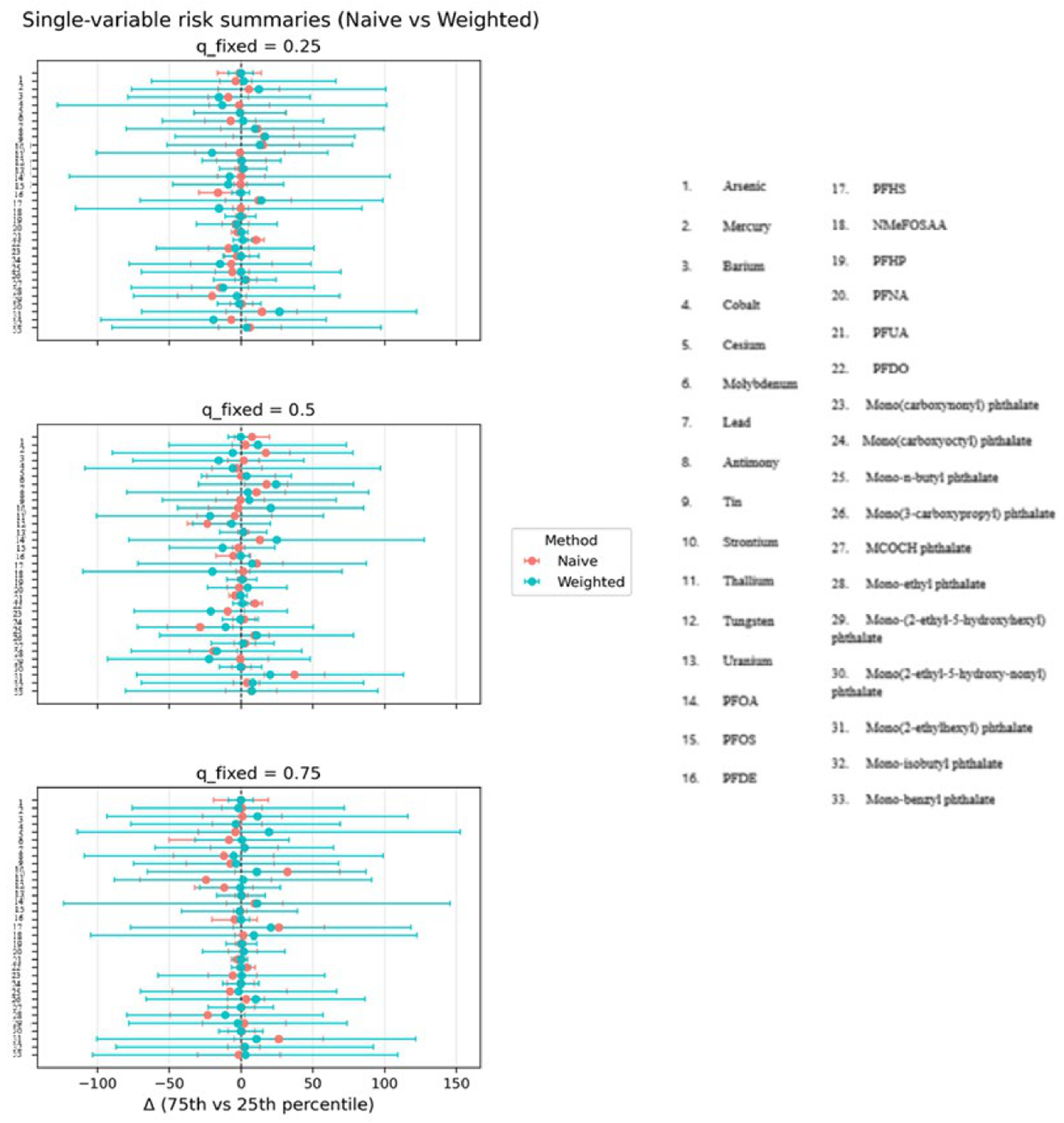
Single-variable risk summaries from BKMR comparing naïve and survey-weighted analyses.

Figure 8 shows single-variable risk summaries from BKMR, comparing naïve and survey-weighted analyses for each exposure in the mixture. Each panel corresponds to a fixed background quantile of the remaining exposures (q_fixed = 0.25, 0.50, and 0.75). The horizontal axis represents the estimated change in the outcome associated with increasing the exposure of interest from its 25th to its 75th percentile. Points indicate posterior mean estimates, and horizontal lines denote 95% intervals; for naïve BKMR these are posterior credible intervals, whereas for survey-weighted BKMR they are replication-based uncertainty intervals. Across exposures, estimated risk differences vary by background quantile, indicating that marginal exposure effects depend on the overall mixture context. In many cases, naïve and weighted estimates are centered near zero; however, the weighted analysis generally shows wider uncertainty intervals, reflecting increased uncertainty after accounting for population weights and clustering. For some exposures, differences between naïve and weighted estimates are more evident at higher background quantiles, suggesting sensitivity of inferred risk contrasts to both mixture composition and survey design adjustment.

**Figure 9.**
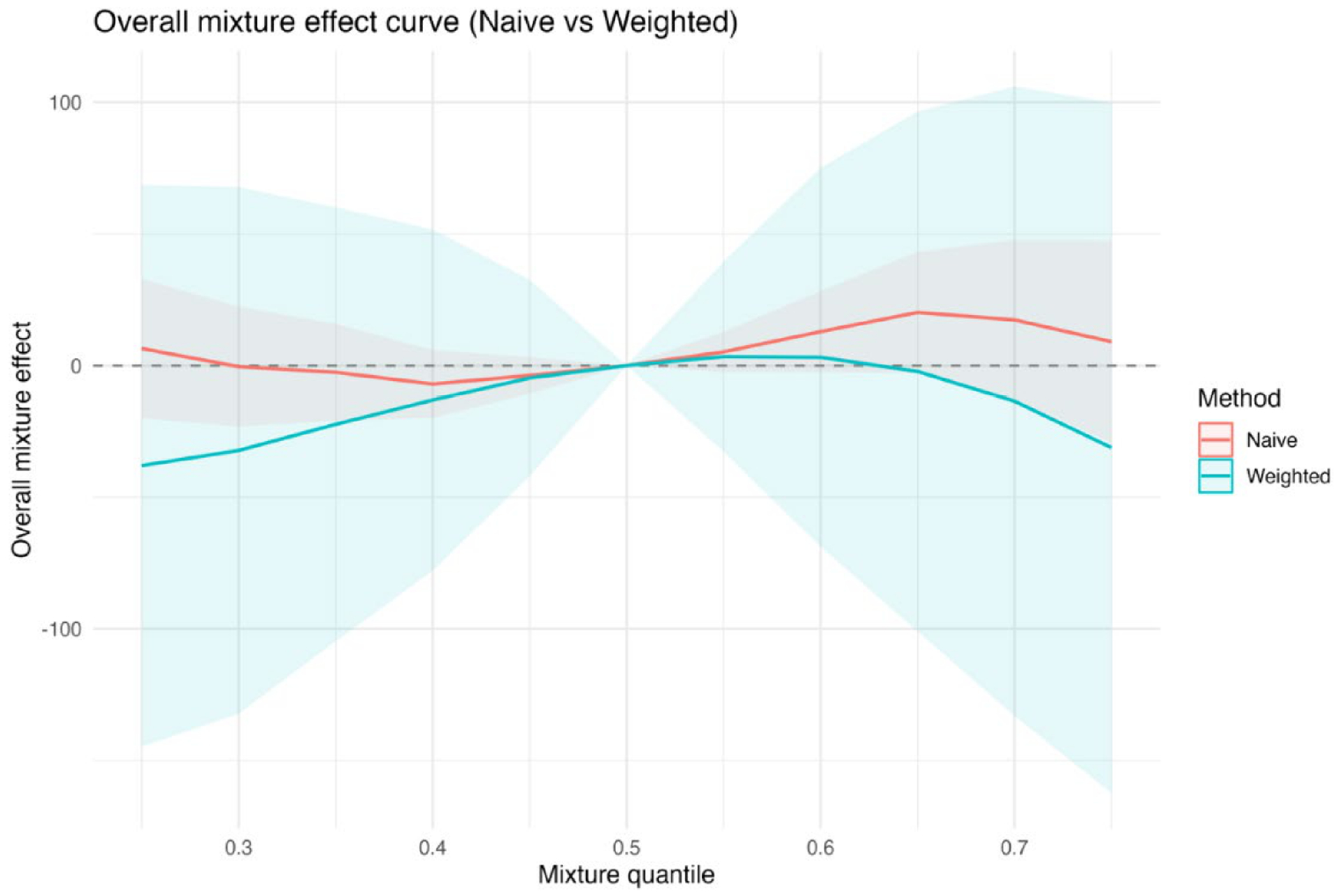
Overall mixture effect curves from BKMR comparing naïve and survey-weighted analyses.

Figure 9 shows the overall mixture effect estimated using BKMR, comparing results from the naïve analysis and the survey-weighted analysis based on PSU bootstrap resampling. The horizontal axis represents the mixture quantile, defined as the joint percentile of all exposures in the mixture, and the vertical axis shows the estimated overall mixture effect relative to the median mixture level. The dashed horizontal line at zero indicates no overall mixture effect. Solid lines represent posterior mean estimates, and shaded regions denote 95% intervals; for naïve BKMR these are posterior credible intervals, whereas for survey-weighted BKMR they are replication-based uncertainty intervals. The mixture effect is evaluated by jointly increasing all exposures across the mixture quantile distribution, with covariates held fixed and the effect at the 50th percentile constrained to zero. At lower mixture quantiles, the naïve analysis suggests modest negative or near-null effects, whereas the weighted analysis indicates greater uncertainty and more negative estimates. At higher quantiles, the naïve analysis shows a gradual increase in the estimated mixture effect, while the weighted analysis exhibits a flatter pattern with substantially wider uncertainty intervals. The broader uncertainty in the weighted analysis, particularly at the distribution tails, reflects the impact of accounting for population weights and clustering on inference for joint exposure effects.

## 4. Discussion

This study presents a practical BKMR-compatible workflow for incorporating key features of complex survey design and evaluates how much survey adjustment affects nonlinear mixture inference. This study addresses a fundamental and often overlooked question in mixture analyses that use population survey data: How much does the complex survey design matter when applying nonlinear mixture models such as BKMR? Using extensive Monte Carlo simulations, we contrasted a naïve, unweighted BKMR fit with a design-aware workflow that integrates survey features through probability-proportional resampling and PSU-level bootstrapping. The simulations systematically varied exposure correlation (ρ), ICC, sample size (*n*), and sampling informativeness, conditions that mirror those encountered in real surveys like NHANES. The estimand of interest was a population-level overall mixture effect, representing the change in the outcome when all exposures jointly shift from their 25th to 75th percentiles [5,41].

Two findings were consistent across scenarios. First, when sampling was informative, naïve BKMR systematically underestimated uncertainty and produced biased estimates, often deviating from the true population effect. This behavior aligns with established survey theory: when inclusion probabilities depend on variables correlated with the outcome, unweighted inference targets the sample rather than the population [2,3,8]. Second, the proposed design-aware workflow substantially improved coverage and reduced bias by realigning inference with the population distribution. Although the intervals widened, this widening was desirable because it represented the additional uncertainty inherent in unequal selection and clustering [4,32]. These improvements persisted even at moderate sample sizes (*n* = 800), reinforcing that design effects cannot be ignored simply because a dataset is large.

The simulation results across metrics and visualizations consistently support these findings. Figure 1 (bias) shows that differences between naïve and design-aware BKMR are small under non-informative sampling but become clear under informative sampling, where naïve estimates depart from the population truth more often. Figures 2–4 show how survey adjustment affects the estimated exposure–response relationships and risk summaries. In particular, the weighted analysis generally shows wider uncertainty intervals and, for some components, attenuated patterns, consistent with carrying forward uncertainty from unequal selection and clustering. Figure 5 (overall mixture effect) similarly shows that the design-aware curve tends to stay closer to zero with broader uncertainty bands, while the naïve curve can show a stronger trend with narrower intervals, an appearance of precision that may not be warranted when the design is ignored. Supplementary Figures S1–S4 summarize performance across simulation conditions for exposures is three, showing that weighted BKMR performed better under informative designs and lower-dimensional exposure mixtures. Functionally, Figures 1–5 reveal that ignoring the survey design produces over-smoothed and sometimes misleading exposure–response surface in higher-dimensional exposure mixtures analysis. The weighted analyses display sharper, more localized nonlinear patterns with wider uncertainty bands that suggest improved alignment with the underlying exposure–response structure. These results demonstrate that survey weighting alters not only the uncertainty of inference but also the shape and interpretation of the learned response surface.

The real-data application further reinforces these conclusions. As shown in Figures 6–9, naïve and survey-weighted BKMR yield broadly similar qualitative exposure–response trends for some exposures, but important differences emerge in effect magnitude, smoothness, and uncertainty. In particular, the survey-weighted analysis consistently produces wider uncertainty intervals and attenuated risk contrasts, reflecting the additional uncertainty induced by unequal selection probabilities and clustering. These patterns closely mirror those observed in the simulation study and demonstrate that the consequences of ignoring survey design are not merely theoretical but arise in practice when analyzing population-representative environmental health data.

The mechanism behind these results is straightforward. Informative sampling alters the composition of the sample by over- or under-representing specific exposure levels. If high-exposure individuals are sampled more frequently, the naïve model effectively learns an exposure–response surface under a biased empirical distribution, leading to distorted contrasts and overconfident intervals. Reweighting or resampling in proportion to the inverse inclusion probabilities corrects this imbalance and restores inference to the population scale [21,23,36,42]. In the absence of native support for survey features within bkmr, our design-aware implementation modifies the data distribution that BKMR uses for inference, allowing it to learn from a population-weighted empirical measure while carrying forward PSU-level uncertainty [4,9,43–45].

Our results also clarify the conditions under which the two approaches converge. When sampling is non-informative and exposure distributions overlap well across strata, the sample represents the population adequately; both methods yield nearly unbiased estimates with close-to-nominal coverage [2]. In contrast, even moderate clustering (ICC = 0.15) amplifies instability under informative designs, leading to overly narrow intervals if PSUs are ignored [24]. Thus, the advantage of the design-aware approach becomes more pronounced as the sampling mechanism becomes more complex or correlated with exposures.

There is an inherent trade-off: incorporating survey design typically produces wider uncertainty intervals. However, as emphasized in survey-sampling theory, this trade-off reflects honest uncertainty, intervals that better account for design effects and clustering [3,46]. From a decision-making standpoint, any apparent precision gained by ignoring the design is illusory and risks overinterpreting spurious patterns.

While our simulations demonstrate improvements over naïve BKMR, we emphasize that the contribution of this paper is the development and evaluation of a BKMR-specific design-aware workflow using currently available software, rather than the introduction of a new general-purpose survey-weighted Bayesian estimator. The framework should therefore be understood as a practical survey-aware approximation for BKMR under current software constraints, rather than as a fully specified pseudo-posterior inferential framework.

### 4.1. Study Limitations

Several limitations should be noted. Although we examined both three- and ten-exposure mixtures in simulation, the simulation framework cannot represent the full range of possible nonlinear exposure–response relationships or dependence structures that may arise in practice. The real-data application using NHANES complements the simulations by illustrating these issues in an applied setting, but it does not remove the inherent constraints of any single simulation design. Nonetheless, across mixture dimensionalities, sampling mechanisms, and levels of clustering, the direction and source of bias from ignoring survey design were consistent, supporting the broader relevance of the design-aware framework [47]. We approximated within-PSU inclusion probabilities and rescaled sampling weights to improve numerical stability while preserving relative inclusion probabilities, an approach commonly used in design-based and Bayesian survey analyses. Although alternative scaling or trimming strategies could be considered, our simulation results indicate that the main conclusions are driven by whether the sampling design is accounted for, rather than by the specific weight normalization used [32]. Uncertainty was estimated using resampled datasets that preserve PSU structure rather than official replicate-weight methods. While alternative replication schemes (e.g., BRR or jackknife) are commonly used for variance estimation in survey analyses, their integration with fully Bayesian, nonlinear models such as BKMR are not straightforward. Our approach provides a practical and theoretically motivated approximation to design-based uncertainty in this setting [4,9].

However, unlike official NHANES replicate-weight methods (e.g., BRR or jackknife), the resampling scheme used here does not exactly reproduce the survey agency’s variance estimation procedure and may not fully capture all design features. The trade-off is that while bootstrap resampling is flexible and compatible with BKMR, official replicate weights remain the preferred approach when available, particularly for design-consistent variance estimation in standard survey analyses.

Computational cost also remains a limitation, as BKMR’s complexity scales with both resampling and mixture dimensionality. Another limitation is the absence of comparisons with other survey-weighted Bayesian or pseudo-posterior approaches, which could provide additional context for evaluating the relative performance of the proposed method. Another limitation is the absence of direct empirical comparison with pseudo-posterior approaches, which incorporate survey weights through likelihood-based adjustments and may provide more theoretically grounded inference under informative sampling. Finally, we acknowledge that medication use, including statins and other lipid-lowering therapies, was not explicitly adjusted for and may influence total cholesterol levels.

### 4.2. Future Directions

Future research should aim to embed survey design features directly into the BKMR likelihood or MCMC algorithm through pseudo-likelihood or pseudo-posterior approaches [21,23,36]. Developing user-friendly interfaces that accept replicate weights and automate posterior combinations would further improve accessibility for applied health researchers. Substantively, design-aware BKMR could be extended to subgroup analyses and richer interaction functionals, aligning more closely with how national health surveys report population-level results. Future work should also include systematic comparisons with alternative survey-aware methods, such as pseudo-posterior approaches and replicate-weight-based estimators, to better understand relative performance under different sampling conditions. In addition, further theoretical work is needed to clarify the conditions under which resampling-based workflows approximate design-consistent inference in nonlinear mixture models.

This work demonstrates that for environmental mixtures estimated from complex surveys, design-aware BKMR is critical when sampling is informative or when populationrepresentative inference is the primary objective. When sampling is informative, naïve BKMR produces biased and overconfident estimates. Incorporating survey weights and PSUs restores population-representative inference and appropriately calibrated uncertainty. The evidence across simulations and figures shows that design-aware BKMR yields more accurate, credible, and interpretable results, offering a practical path forward for mixture modeling in large-scale health surveys.

## 5. Conclusions

In our Monte Carlo study, naïve BKMR performed poorly whenever sampling was informative or data were clustered: point estimates were biased and nominal 95% intervals systematically under-covered. In contrast, a design-aware approach based on PSU bootstrapping and weight-proportional resampling markedly improved coverage and reduced bias, although at the expected cost of wider but more honest uncertainty intervals. Although the design-aware approach improves empirical coverage relative to naïve BKMR, coverage remains below nominal levels in several scenarios. This indicates that while accounting for complex survey design reduces bias and undercoverage, the proposed method does not fully resolve inferential limitations associated with weighted mixture modeling. When sampling was non-informative and overlap was good, naïve and weighted results were largely consistent, suggesting that the benefits of design-aware inference are most pronounced in realistic survey settings. These conclusions were also reflected in the NHANES application, where naïve and survey-weighted BKMR showed differences in effect magnitude, smoothness, and uncertainty. In that applied example, the survey-weighted analysis generally produced wider intervals and more attenuated patterns, consistent with the additional uncertainty introduced by unequal selection probabilities and clustering. Because the NHANES analysis is presented as a demonstrative application rather than a fully inferential analysis, the primary methodological conclusions of this study are based on the simulation results. Given that national health surveys routinely employ complex sampling designs, we recommend that analysts consider design-aware BKMR when population-representative inference is the objective and interpret naïve fits as secondary or exploratory analyses.

## Supplementary Material

Supplementary

The following supporting information can be downloaded at: https://www.mdpi.com/article/10.3390/stats9030046/s1, Figure S1. Univariate exposure–response functions from BKMR; Figure S2: Bivariate exposure–response functions: design-aware; Figure S3. Single-exposure risk from naïve and survey-weighted BKMR under different conditioning scenarios; Figure S4. Overall mixture effects across exposure quantiles estimated using naïve and survey-weighted BKMR.

## Figures and Tables

**Table 1.** Posterior inclusion probabilities for a three-exposure mixture (Z1–Z3).

Exposure	Groups	Conditional PIP (Naïve)	Group PIP (Naïve)	Conditional PIP (Weighted)	Group PIP (Weighted)
Z1	1	1	1	1	1
Z2	2	1	1	1	1
Z3	3	1	1	1	1

**Table 2.** Posterior inclusion probabilities for a ten-exposure mixture (Z1–Z10).

Exposure	Group	Conditional PIP (Naïve)	Group PIP (Naïve)	Conditional PIP (Weighted)	Group PIP (Weighted)
Z1	1	1	1	1	1
Z2	2	1	1	1	1
Z3	3	1	1	1	1
Z4	4	0.882	0.882	0.993	0.993
Z5	5	0.135	0.135	0.992	0.992
Z6	6	0.122	0.122	0.819	0.819
Z7	7	0.118	0.118	1	1
Z8	8	0.121	0.121	0.993	0.993
Z9	9	0.042	0.042	0.991	0.991
Z10	10	0.237	0.237	0.975	0.975

**Table 3.** Baseline environmental exposure levels of 4345 participants from the 2013–2014 cycle of NHANES.

Chemical Class (Analytes Included)	*n*	Mean (SD)
Metals		
Arsenic (As, μg/L)	4345.0	15.88 (30.82)
Mercury (Hg, μg/L)	4345.0	0.54 (1.69)
Barium (Ba, μg/L)	4345.0	1.78 (2.33)
Cobalt (Co, μg/L)	4345.0	0.64 (1.47)
Cesium (Cs, μg/L)	4345.0	4.91 (2.38)
Molybdenum (Mo, μg/L)	4345.0	51.99 (40.52)
Lead (Pb, μg/dL)	4345.0	0.47 (0.62)
Antimony (Sb, μg/L)	4345.0	0.07 (0.11)
Tin (Sn, μg/L)	4345.0	1.33 (3.80)
Strontium (Sr, μg/L)	4345.0	122.33 (152.25)
Thallium (TI, μg/L)	4345.0	0.18 (0.10)
Tungsten (W, μg/L)	4345.0	0.12 (0.20)
Uranium (U, μg/L)	4345.0	0.01 (0.03)
PFAS		
Perfluorooctanoic acid (PFOA) (ng/mL)	4345.0	2.30 (2.19)
Perfluorooctanesulfonic acid (PFOS) (ng/mL)	4345.0	8.15 (24.00)
Perfluorodecanoic acid (PFDE) (ng/mL)	4345.0	0.30 (1.00)
Perfluorohexanesulfonic acid (PFHS) (ng/mL)	4345.0	1.95 (1.70)
Perfluorononanoic acid (PFNA) (ng/mL)	4345.0	0.87 (0.63)
N-Methyl perfluorooctane sulfonamidoacetic acid (NMeFOSAA)	4345.0	0.18 (0.22)
Perfluoroheptanoic acid (PFHP)	4345.0	0.08 (0.04)
Perfluoroundecanoic acid (PFUA)	4345.0	0.22 (1.58)
Perfluorododecanoic acid (PFDO)	4345.0	0.09 (0.13)
Phthalates		
Mono-ethyl phthalate (MEP) (ng/mL)	4345.0	204.46 (874.78)
Mono-n-butyl phthalate (MnBP) (ng/mL)	4345.0	17.45 (20.07)
Mono(2-ethylhexyl) phthalate (MEHP) (ng/mL)	4345.0	2.38 (3.99)
Mono-isobutyl phthalate (MiBP) (ng/mL)	4345.0	14.06 (17.01)
Mono-benzyl phthalate (MBzP) (ng/mL)	4345.0	9.93 (14.49)
Mono-(2-ethyl-5-carboxypentyl) phthalate (ng/mL)	4345.0	0.11 (0.23)
Mono-(2-ethyl-5-hydroxyhexyl) phthalate (ng/mL)	4345.0	12.05 (21.97)
Mono-(2-ethyl-5-hydroxynonyl) phthalate (ng/mL)	4345.0	0.62 (2.73)
Mono-(3-carboxypropyl) phthalate (MCPP) (ng/mL)	4345.0	5.17 (11.58)
Mono (carboxynonyl) phthalate (ng/mL)	4345.0	6.02 (21.15)
Mono(carboxyoctyl) phthalate (MCOCH) (ng/mL)	4345.0	53.34 (85.20)

**Table 4.** Posterior inclusion probabilities (PIPs) for individual exposures and exposure groups in the real data analysis.

Exposure	Group	Conditional Naïve	Group PIP Naïve	Conditional Weighted	Group PIP Weighted
Antimony	1	0.848	1.00	1.00	1.00
Arsenic	1	0.789	1.00	0.707	1.00
Barium	1	0.96	1.00	1.00	1.00
Cesium	1	0.851	1.00	1.00	1.00
Cobalt	1	1.00	1.00	1.00	1.00
Lead	1	1.00	1.00	1.00	1.00
Mercury	1	0.925	1.00	1.00	1.00
Molybdenum	1	0.837	1.00	1.00	1.00
Strontium	1	0.968	1.00	1.00	1.00
Thallium	1	0.928	1.00	1.00	1.00
Tin	1	0.981	1.00	1.00	1.00
Tungsten	1	0.976	1.00	1.00	1.00
Uranium	1	0.984	1.00	0.925	1.00
NMeFOSAA	2	0.992	1.00	1.00	1.00
PFDE	2	1.00	1.00	1.00	1.00
PFDO	2	1.00	1.00	1.00	1.00
PFHP	2	1.00	1.00	0.901	1.00
PFHS	2	0.939	1.00	1.00	1.00
PFNA	2	1.00	1.00	0.981	1.00
PFOA	2	1.00	1.00	1.00	1.00
PFOS	2	1.00	1.00	1.00	1.00
PFUA	2	1.00	1.00	0.960	1.00
Mono-ethyl phthalate (MEP)	3	1.00	1.00	1.00	1.00
Mono-n-butyl phthalate (MnBP)	3	0.949	1.00	1.00	1.00
Mono(2-ethylhexyl) phthalate (MEHP)	3	0.946	1.00	1.00	1.00
Mono-isobutyl phthalate (MiBP)	3	1.00	1.00	1.00	1.00
Mono-benzyl phthalate (MBzP)	3	0.925	1.00	1.00	1.00
Mono-(2-ethyl-5-carboxypentyl) phthalate	3	0.984	1.00	1.00	1.00
Mono-(2-ethyl-5-hydroxyhexyl) phthalate	3	0.930	1.00	1.00	1.00
Mono-(2-ethyl-5-hydroxynonyl) phthalate	3	0.987	1.00	1.00	1.00
Mono-(3-carboxypropyl) phthalate (MCPP)	3	0.771	1.00	1.00	1.00
Mono (carboxynonyl) phthalate	3	0.901	1.00	1.00	1.00
Mono(carboxyoctyl) phthalate (MCOCH)	3	0.877	1.00	0.835	1.00

## Data Availability

The NHANES dataset is publicly available online at https://www.cdc.gov/nchs/nhanes/ (accessed on 5 March 2026). Simulation code and analysis scripts, including full implementation details, parameter settings are publicly available at https://github.com/Doreennolda/Incorporating-Complex-Survey-Design-in-BKMR (accessed on 31 March 2026).
