# Supplementary material for: A Practical Framework for Incorporating Complex Survey Design in Bayesian Kernel Machine Regression
Source: Stats (Basel). Author manuscript; Available in PMC 2026 Jun 1. (PMC13220982; doi:10.3390/stats9030046)
Supplement: Supplementary [file NIHMS2172665-supplement-Supplementary.pdf]

Supplementary materials

Figures for 3 exposures

Figure S1 presents the estimated univariate exposure–response functions for the three-exposure setting, comparing naïve BKMR with survey-weighted BKMR that accounts for the complex sampling design. For each exposure, the curves depict the posterior mean of the exposure–response function, holding the remaining exposures fixed at their median values. The shaded bands represent 95% intervals; for naïve BKMR these are posterior credible intervals, whereas for survey-weighted BKMR they represent replication-based uncertainty intervals. Across the three exposures, both approaches indicate nonlinear relationships with the outcome, although the weighted analysis generally shows wider uncertainty intervals and some attenuation in the estimated response patterns after accounting for unequal selection probabilities and clustering. These results are consistent with the broader simulation findings and are presented as a lower-dimensional complement to the main figures.

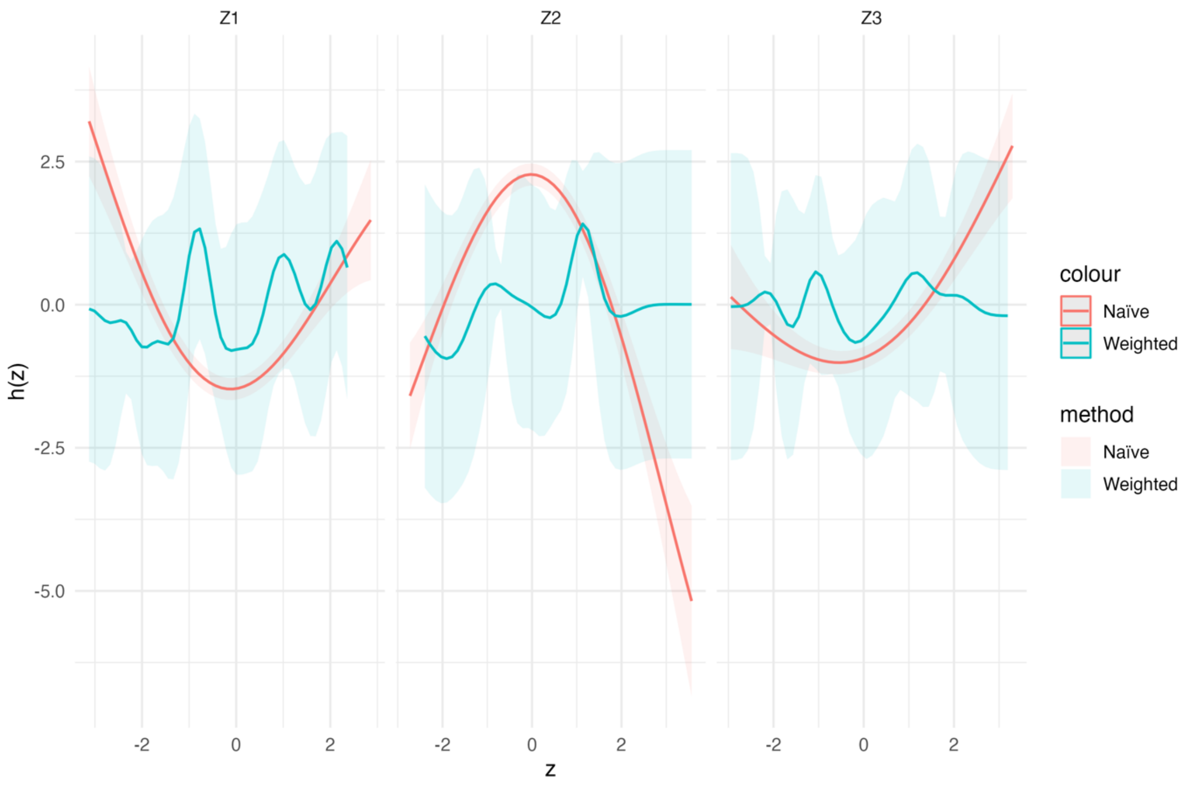

Figure S1. Univariate exposure–response functions for the three-exposure setting, comparing naïve and survey-weighted BKMR.

Figure S2 presents bivariate exposure–response functions for the three-exposure setting, comparing naïve and survey-weighted BKMR. Each panel shows the relationship between a focal exposure and the outcome, conditional on the second exposure fixed at its 25th, 50th, or

75th percentile, while the remaining exposure is held at its median value. As in the main analysis, variation across conditioning quantiles suggests potential nonlinear interaction effects within the mixture. In this three-exposure setting, the naïve analysis tends to show greater curvature and wider separation between quantile-specific curves, whereas the survey-weighted analysis generally exhibits smoother response patterns with increased uncertainty after accounting for unequal selection probabilities and clustering. These differences illustrate how complex survey design can influence the apparent shape and strength of estimated interaction patterns.

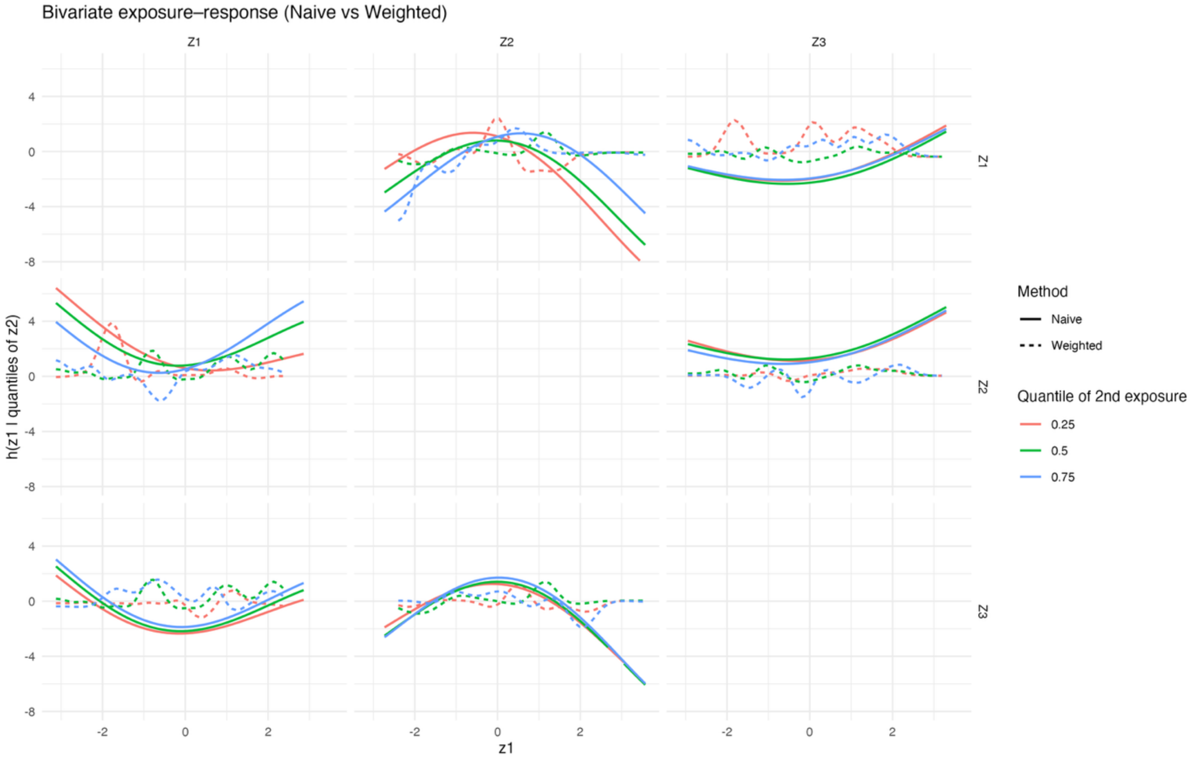

Figure S2. Bivariate exposure–response functions for the three-exposure setting, comparing naïve and survey-weighted BKMR.

Figure S3 presents the single-variable risk summaries for the three-exposure setting, comparing naïve and survey-weighted BKMR. For each exposure, the plotted point estimate represents the estimated change in the outcome when that exposure increases from the 25th to the 75th percentile, while the remaining exposures are fixed at the 25th, 50th, or 75th percentile. Horizontal bars denote 95% intervals; for naïve BKMR these are posterior credible intervals, whereas for survey-weighted BKMR they are replication-based uncertainty intervals. Across conditioning levels, the estimated risk differences are generally more attenuated in the survey-weighted analysis and are accompanied by wider uncertainty intervals. These patterns are consistent with the main simulation results, where accounting for complex survey design primarily affected uncertainty and, in some cases, the apparent magnitude of marginal risk contrasts.

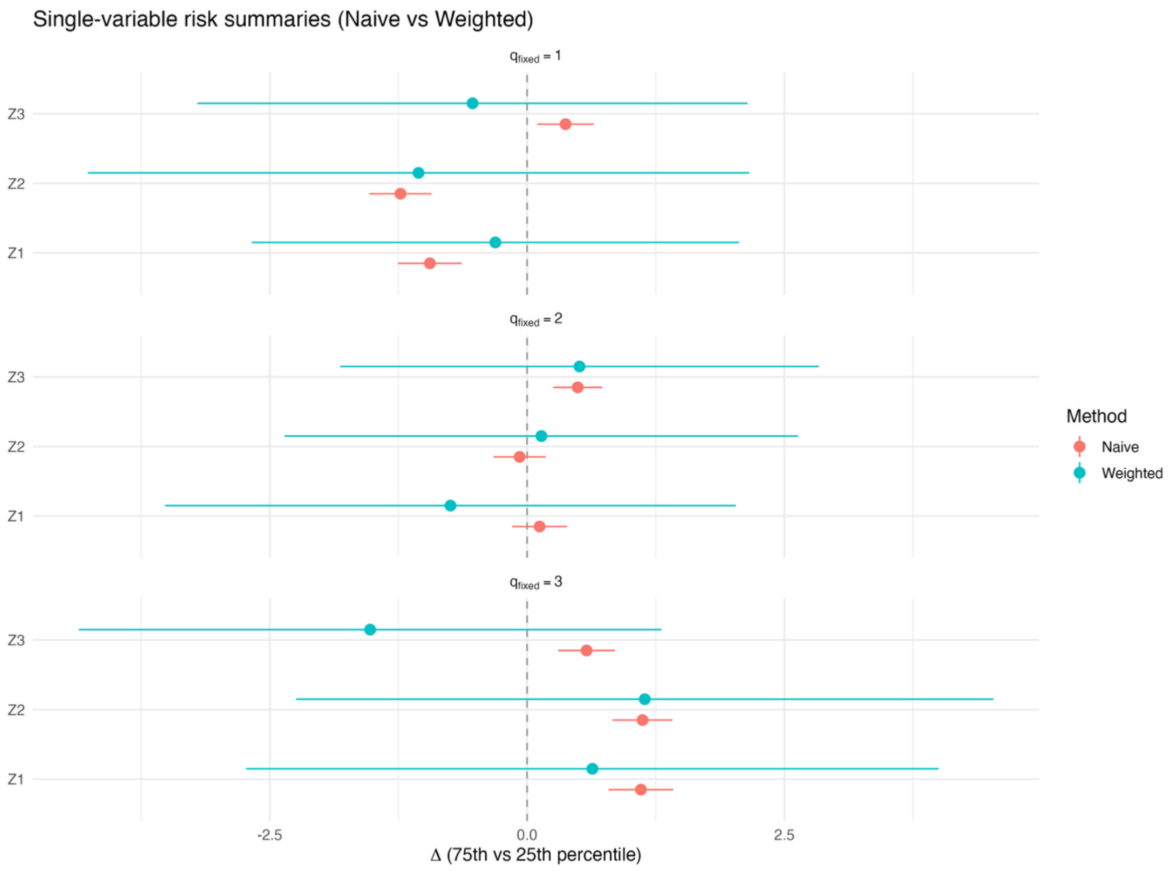

Figure S3. Single-variable risk summaries for the three-exposure setting from naïve and survey-weighted BKMR under different conditioning scenarios.

Figure S4 presents the estimated overall BKMR mixture effect for the three-exposure setting, comparing naïve and survey-weighted analyses across exposure quantiles. Each curve represents the estimated change in the outcome when all exposures are jointly set to a given quantile, relative to the median exposure level. Shaded bands denote 95% intervals; for naïve BKMR these are posterior credible intervals, whereas for survey-weighted BKMR they are replication-based uncertainty intervals. Consistent with the main simulation results, the survey-weighted analysis tends to remain closer to zero and is accompanied by wider uncertainty intervals across quantiles, reflecting the additional variability introduced by accounting for unequal selection probabilities and clustering. In contrast, the naïve analysis shows a stronger upward trend at higher quantiles with comparatively narrower intervals,

suggesting greater apparent precision when the complex survey design is ignored.

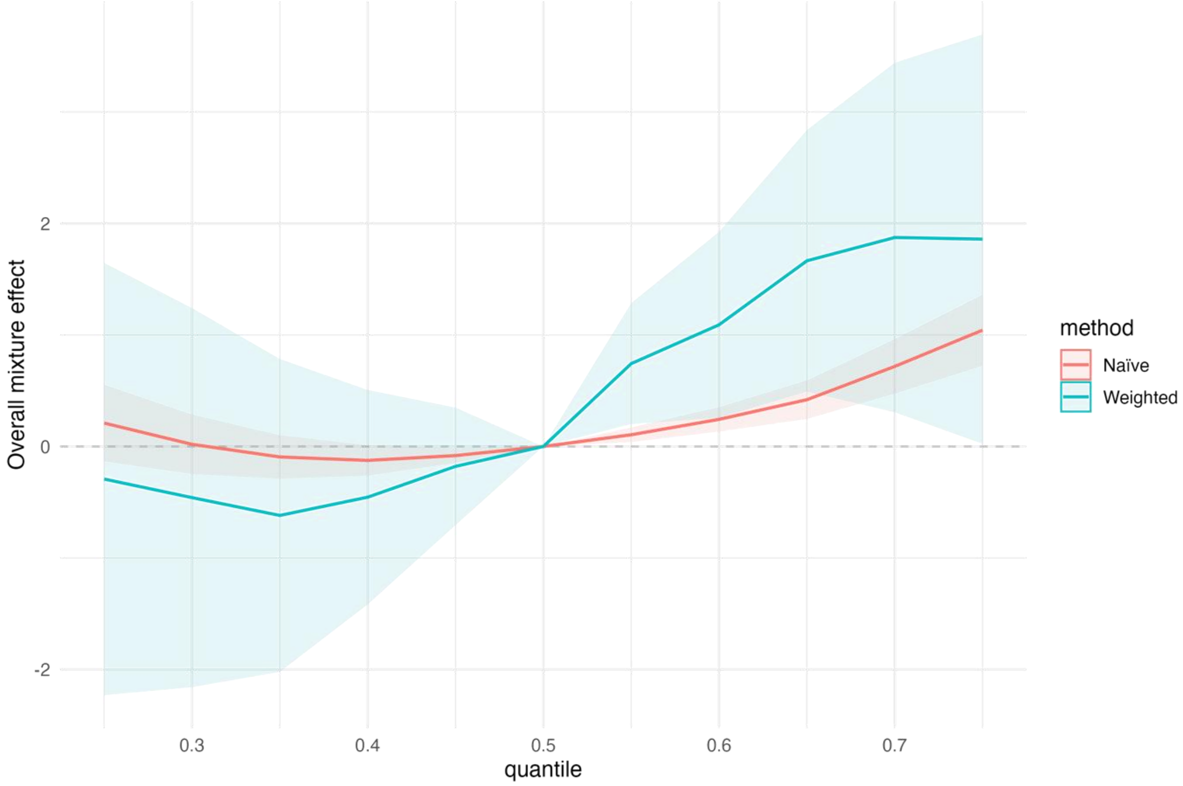

Figure S4. Overall mixture effect by exposure quantile for the three-exposure setting, comparing naïve and survey-weighted BKMR.

ICC Calibration in the Simulation Design

To induce within-cluster dependence, the simulation model included a cluster-level random intercept. Specifically, for individual i in cluster c, the outcome was generated as

$$Y_{ic} = h(Z_{ic}) + u_c + \varepsilon_{ic},$$

where $h(Z_{ic})$ denotes the exposure-response function, $u_c \sim \mathcal{N}(0, \sigma_u^2)$ is the cluster-level random effect, and $\varepsilon_{ic} \sim \mathcal{N}(0, \sigma_e^2)$ is the individual-level error term. The cluster effect u_c induces correlation among individuals sampled from the same PSU.

The target intra-class correlation coefficient (ICC) was defined as

$$\text{ICC} = \frac{\sigma_u^2}{\sigma_u^2 + \sigma_e^2},$$

In all simulations, the individual-level error variance was fixed at σ_e^2= 1. For each target ICC scenario, the cluster-level variance was calibrated as

$$\sigma_u^2 = \frac{\mathrm{ICC}}{1 - ICC}\sigma_e^2$$

Thus, the variance values used in the simulations were:

- **ICC = 0.00:** σ_u^2 = 0.00
- **ICC = 0.15:** σ_u^2 = 0.15/0.85 = 0.1765

These values were used when generating the PSU-level random effects prior to sampling. When ICC = 0, no between-PSU dependence was induced. When ICC = 0.15, moderate within-PSU correlation was introduced, reflecting realistic clustering often observed in complex survey settings. This calibration ensured that the simulated outcomes had the intended dependence structure before stratified two-stage sampling and informative within-cluster selection were applied.
